# The Post-Transcriptional Regulatory Protein CsrA Amplifies Its Targetome through Direct Interactions with Stress-Response Regulatory Hubs: The EvgA and AcnA Cases

**DOI:** 10.3390/microorganisms12040636

**Published:** 2024-03-22

**Authors:** Alejandra Matsuri Rojano-Nisimura, Kobe B. Grismore, Josie S. Ruzek, Jacqueline L. Avila, Lydia M. Contreras

**Affiliations:** 1 Department of Molecular Biosciences, The University of Texas at Austin, 100 East 24th St. Stop A5000, Austin, TX 78712, USA; matsuri.rojano@utexas.edu; 2 McKetta Department of Chemical Engineering, The University of Texas at Austin, 200 E. Dean Keeton St. Stop C0400, Austin, TX 78712, USA; kobegrismore@utexas.edu (K.B.G.); josieruzek@utexas.edu (J.S.R.); jacqueline.le.avila@gmail.com (J.L.A.)

**Keywords:** CsrA, Csr network, post-transcriptional regulation, RNA–protein interactions, bacterial stress responses

## Abstract

Global rewiring of bacterial gene expressions in response to environmental cues is mediated by regulatory proteins such as the CsrA global regulator from *E. coli.* Several direct mRNA and sRNA targets of this protein have been identified; however, high-throughput studies suggest an expanded RNA targetome for this protein. In this work, we demonstrate that CsrA can extend its network by directly binding and regulating the *evgA* and *acnA* transcripts, encoding for regulatory proteins. CsrA represses EvgA and AcnA expression and disrupting the CsrA binding sites of *evgA* and *acnA*, results in broader gene expression changes to stress response networks. Specifically, altering CsrA-*evgA* binding impacts the genes related to acidic stress adaptation, and disrupting the CsrA-*acnA* interaction affects the genes involved in metal-induced oxidative stress responses. We show that these interactions are biologically relevant, as evidenced by the improved tolerance of *evgA* and *acnA* genomic mutants depleted of CsrA binding sites when challenged with acid and metal ions, respectively. We conclude that EvgA and AcnA are intermediate regulatory hubs through which CsrA can expand its regulatory role. The indirect CsrA regulation of gene networks coordinated by EvgA and AcnA likely contributes to optimizing cellular resources to promote exponential growth in the absence of stress.

## 1. Introduction

Post-transcriptional regulatory networks allow bacteria to rapidly adapt and respond to changing environments. In *Gammaproteobacteria*, the carbon storage regulatory protein A (CsrA) acts as a pleiotropic regulator of gene expression, particularly impacting central carbon metabolism [1,2]. CsrA is an RNA-binding protein (RBP), part of the Csr network, that controls multiple cellular processes. Our current understanding of CsrA-mediated regulation supports the notion that CsrA represses stationary-phase processes (such as biofilm formation, iron storage, metabolism of alternative carbon sources, and stress responses) and activates pathways related to exponential growth (motility, glycolysis, and virulence) [3].

Mechanistically, CsrA post-transcriptionally regulates gene expression by directly binding to target RNAs, altering their translation and/or transcript stability. In the case of sRNAs, it can additionally impact their interactions with cognate RNAs [4]. Molecularly, CsrA-binding typically involves two hairpin-looped regions containing a high-affinity “GGA” triplet sequence (herein referred to as GGA motif) [5]. Notably, CsrA has also been observed to bind at non-consensus ANGGN-like sites which contribute (to a smaller extent) to target regulation [4,6]. To date, there are 29 confirmed direct CsrA RNA targets in *E. coli* [4,6,7,8,9,10,11,12,13,14,15,16,17,18,19,20,21]. In this paper, we define a confirmed direct target as an RNA for which a binding interface has been fully mapped (via in vitro footprinting and/or mutational analysis of CsrA binding sites) and/or a direct physical interaction has been experimentally confirmed (typically by in vitro binding assays). In addition, there are 92 functional targets for which CsrA-mediated regulation has been observed in reporter assays (Supplementary Table S1), albeit it is yet to be determined whether those regulatory effects occur through direct or indirect interactions [22,23]. Moreover, large-scale studies have identified over 500 other transcripts as potential CsrA targets by computational predictions, transcriptomics, and/or CLIP-based approaches, suggesting that this RNA-binding protein may exert broader regulatory effects than what has been characterized so far [2,11,22,24,25,26]. The true direct targetome and the mechanisms by which global regulatory proteins like CsrA influence their targets, however, continue to be debatable; a major challenge is that these are, potentially, highly dynamic regulatory interactions that can vary widely under different cellular and environmental conditions and, as such, are difficult to detect under one specific experimental condition. The extent of the CsrA regulatory network is of particular interest in the RNA field since studies of this major post-transcriptional network are instructive in our broad understanding of molecular interactions that underlie the versatility of RNA–protein regulation [23,27,28,29].

In this work, we characterize two mRNA targets that directly interact with the CsrA protein: *evgA* and *acnA*. EvgA is a transcriptional activator part of the EvgS/EvgA two-component system. EvgS is a sensor kinase that responds to changes in pH and the presence of monovalent cations (Na+, K+) [30], although the details regarding its sensing mechanism are still unknown. EvgA is activated by EvgS via phosphorelay, and it subsequently activates genes important for acid resistance, osmotic adaptation, and antibiotic resistance. Specifically, DNA microarrays identified genes of the acid resistance 2 (AR2) system, high osmolarity-induced genes, and genes encoding for multidrug efflux pumps as upregulated by the overproduction of EvgA [31]. Regarding AcnA, the apoform of this protein functions as an RBP that acts as a survival enzyme during nutritional and oxidative stress [32,33]. AcnA binds to the 3′ UTR of the *acnA* transcript preventing its degradation and increasing its translation, which provides a positive feedback mechanism to rapidly respond to stress [33]. Protein expression profiles using mutant strains revealed that AcnA enhances the production of additional anti-oxidative stress proteins such as SodA and TrxB, although *sodA* is the only transcript for which AcnA-mediated regulation has been characterized to date [34].

In this study, we characterize the interactions between CsrA and the mRNAs encoding for AcnA and EvgA using a mutational approach in combination with in vitro binding assays and in vivo reporter assays. Importantly, we show through RNA-seq and phenotypic assays that *evgA* and *acnA* represent intermediate regulatory hubs through which CsrA can expand its regulatory effects. We show that CsrA can influence acidic stress tolerance through its direct interaction with *evgA* and pathways related to metal ion-induced oxidative and osmotic stresses (DNA repair, amino acid biosynthesis, lipid biosynthesis, breakdown of oxygen radicals, and iron–sulfur cluster formation, among others) through its interaction with *acnA*. 

Altogether, our results exemplify how the direct interaction between CsrA and mRNAs encoding for intermediate regulatory proteins, like EvgA and AcnA, allow CsrA to serve as a broad root node of regulation for bacterial gene expression and to have higher global reach in reshaping cellular behavior and controlling diverse physiological processes.

## 2. Materials and Methods

### 2.1. Plasmids and Strains

All *E. coli* strains used in this study are K-12 MG1655 derivatives and are listed in Supplementary Table S3. A detailed description of plasmids and the cloning strategy used to generate them can be found in Supplementary Table S4. Oligonucleotide primers and double-stranded DNA fragments used for cloning and as templates for in vitro transcription of RNA fragments were purchased from Integrated DNA Technologies (IDT; Coralville, IA, USA) and are included in Supplementary Table S5. Plasmid sequences were verified by long-read sequencing (Plasmidsaurus; Eugene, OR, USA). Genomic insertions were confirmed by PCR amplification followed by long-read amplicon sequencing (Plasmidsaurus; Eugene, OR, USA).

The original pUC19-T7link-sfGFP parent plasmid used in the cloning of the DNA template plasmids for in vitro transcription–translation assays was a generous gift from Dr. Svetlana Harbough and the AFRL team.

The *evgA* and *acnA* genomic mutants in which CsrA binding sites were mutated to eliminate CsrA-binding were generated as described in [4] using a CRISPR-*cas9* genome modification protocol originally developed by Mehrer et al. (2015) [35]. Briefly, a two-plasmid system, generously provided by the lab of Dr. Brian Pfleger, was used to genomically insert a dsDNA sequence with the desired mutation in place of the genomic copy of *evgA* or *acnA* via *cas9*-mediated cleavage and λ-red recombination. 

### 2.2. In Vitro Electrophoretic Mobility Shift Assays

RNA sequences for *evgA*, *acnA*, and their respective mutants were transcribed using the MEGA Script IVT Kit (Thermo Fisher Scientific; Waltham, MA, USA) following the manufacturer’s instructions. Double-stranded DNA templates were removed via DNase I digestion, and the quality of the transcripts was assessed on 8% urea gels stained with Sybr Green II (Thermo Fisher Scientific; Waltham, MA, USA). Afterward, 5′-end labeling was conducted using T4 Polynucleotide Kinase (NEB) and [gamma-32P] ATP (PerkinElmer; Waltham, MA, USA). RNAs were purified using DTR Gel Filtration Cartridges (EdgeBio; Gaithersburg, MD, USA), and their concentration was measured via fluorometric quantification using the Qubit RNA High Sensitivity Kit (Thermo Fisher Scientific; Waltham, MA, USA).

EMSAs were performed as described in [4]. The concentrations of CsrA were selected to mimic the protocol detailed in [36] with additional CsrA concentrations to expand the range in which a CsrA-RNA bound complex was initially observed to form. Radiolabeled RNAs were incubated with increasing concentrations of CsrA (0–300 nM) for 30 min prior to loading and running on a 10% non-denaturing polyacrylamide gel with 0.5× TBE running buffer (IBI Scientific; Dubuque, IA, USA; 10× composition: 89 mM Tris, 89 mM Boric Acid, 2 mM EDTA) at 170 V for 6–8 h at 4 °C. Gels were exposed overnight on phosphor-imaging cassettes (bioWORLD; Dublin, OH, USA) and imaged on a Typhoon FLA 700 (GE Health Life Science; Chicago, IL, USA) at 1000 V. 

### 2.3. In Vivo Fluorescence Reporter Assays

Translational reporter assays were performed according to previously published methods using this system [22,37,38] with some modifications. Briefly, mRNA leader-*gfp* fusion plasmids were designed using pHL1756 as a parent plasmid (Addgene #53036). A detailed description of the cloning strategy is included in Supplementary Table S4. These plasmids were paired with a second plasmid, pHL600 (Addgene #37563), which contains *csrA* expression under the control of an IPTG-inducible promoter. The two-plasmid system was expressed in *E. coli* MG1655 K-12 Δ*csrABCD* Δ*pgaABCD* Δ*glgCAP* (herein referred to as Csr system deletion strain). Single colonies were picked and grown overnight in 5 mL of LB media supplemented with 50 μg/mL kanamycin (Amresco; Solon, OH, USA) and 100 μg/mL carbenicillin (Grainger; Lake Forest, IL, USA). Afterward, the cells were seeded (1:100 dilution) in 30 mL of fresh media and grown at 37 °C and 120 rpm until they reached the target, OD_600_~0.4. At that point, the cultures were split in half and 1 mM IPTG was added to a 15 mL volume of culture to induce CsrA expressions. After two additional hours of growth, green fluorescence was measured with a BD LSRFortessa flow cytometer (~20,000 cells per sample). The final OD_600_ values were ~1.5–2.0. Fold changes in median green fluorescence between +CsrA and −CsrA conditions were determined for each biological replicate. To evaluate the statistical significance, the fluorescence values for each mRNA variant tested were contrasted to those of the negative controls by a heteroscedastic unpaired *t*-test (*p*-value ≤ 0.05).

### 2.4. In Vitro Coupled Transcription-Translation Assays

Coupled transcription–translation assays were conducted using the PURExpress kit (NEB; Ipswich, MA, USA) as described in [39] with a few modifications. Concretely, template DNA plasmids contained a T7 promoter driving the transcription of *evgA-gfp, acnA-gfp*, or their relevant mutants. Reaction mixtures containing 4.25 nM plasmid DNA template and various amounts of purified CsrA (0–2000 nM) were incubated for 4 h at 30 °C. GFP fluorescence was monitored every 15 min in a Cytation3 plate reader instrument. To evaluate the regulatory effects of CsrA, fluorescence values obtained for the reactions containing CsrA (125–2000 nM) were compared to those of the reactions without CsrA (0 nM) by a heteroscedastic unpaired *t*-test. Values with *p*-value ≤ 0.05 were considered statistically significant, indicating that CsrA addition was causing a regulatory effect in the translation of the fusion construct. 

### 2.5. RNA Isolation and Transcriptomics Analysis

Total RNA was extracted from the cultures of biological triplicates grown in LB to the desired OD_600_ (0.8 for the Early Exponential condition, 1.5 for the Late Exponential condition, and 2.5 for Stationary; Supplementary Figure S5). Approximately 2 × 10^9^ cells per strain and condition were treated with 200 µL of lysozyme buffer (2 mg/mL, Thermo Fisher Scientific; Waltham, MA, USA) to allow for cell lysis. Afterward, the cells were spun down, and the supernatant was used for RNA clean-up and purification using the Quick DNA/RNA Miniprep Plus Kit (Zymo Research; Orage, CA, USA) and following the manufacturer’s instructions. RNA was treated with DNase I (NEB; Ipswich, MA, USA) and assessed for quality using a Bioanalyzer (Agilent; Santa Clara, CA, USA), with all samples having an RNA Integrity Number (RIN) of >9. RNA was submitted to the Center for Biomedical Research Support at the University of Texas at Austin for rRNA depletion, library preparation, and Illumina-based RNA sequencing (Illumina NovaSeq SP platform). All samples were depleted of rRNA before sequencing.

Raw RNA reads were processed using the resources of the Texas Advanced Computing Center (TACC) using previously described pipelines [22,40]. Following reads alignment, counting, and normalization, differential expression was determined using the DESeq2 package [41]. Contrasting each genomic mutant strain to the wild-type strain, the differential expression of a transcript was considered significant when, while using a Wald test, the P_adj_ value was <0.05 and the log2 fold-change was ≥0.75. 

### 2.6. Network Reconstruction and Analysis

Cluster networks were generated using the Cytoscape apps geneMANIA and clustermaker2 and visualized in Cytoscape version 3.10.1 [42]. Gene names and their respective log2 fold-change for Early Exponential, Late Exponential, and Stationary growth phases from the differential expression dataset were imported into geneMANIA to create associations between genes. Associations considered in geneMANIA include known co-expression, genetic interaction, physical interaction, and shared protein domains for each gene. The top-related genes were determined using automatic weighting. Clustermaker2 was used to cluster genes based on known functional relationships. The parameters used to create the cluster maps were a degree cutoff of 2, a “haircut” filter, a node cutoff score of 0.2, a k-core value of 2, a max depth of 100, and restoring inter-cluster edges after layout. Within each figure generated using geneMANIA and clustermaker2, each node on the cluster map represents a gene imported from the differential expression data or a gene inserted by Cytoscape during network reconstruction. The size of each node represents the number of growth conditions under which the gene of interest was found to be differentially expressed. Genes with similar functions were filled with the same color, while genes clustered together were outlined with the same color. Genes with an unknown function are filled in gray, while genes not in a cluster have a thin black outline.

## 3. Results

### 3.1. CsrA Binds Directly to the Leader Sequences of the evgA and acnA Transcripts Encoding Regulatory Proteins

The regulatory impact of CsrA on 92 functional RNA targets was demonstrated by Sowa et al. (2017) [22] and Leistra et al. (2018) [23] using in vivo translational reporter assays, although direct CsrA-binding was not evaluated in those works. Two interesting observations from these works are as follows: (i) approximately 20% of CsrA functional targets, identified via reporter assays, encode for regulatory proteins (i.e., transcription factors, RNA-binding proteins, post-translational-modification proteins, etc.) and (ii) for these regulatory proteins, several of their known targets were identified as genes that are functionally impacted by the cellular presence of CsrA, which could suggest complex regulatory networks involving both direct and indirect CsrA targets (Supplementary Table S1). Specifically, this interplay suggested to us that, for some of the 92 functional RNA targets, CsrA regulation could be potentially impacted indirectly by the direct binding of CsrA to mRNAs that encode for their known regulatory partners, which could be acting as “intermediate regulatory hubs”. Here, we define an intermediate regulatory hub as a central node that broadens the number of genes that CsrA can indirectly influence and allows for the fine-tuning of bacterial regulatory networks in response to external cues. In that sense, an intermediate regulatory hub, as it pertains to the CsrA regulon, is a regulatory protein with known downstream targets that have been identified as functionally affected by CsrA (presumably due to indirect effects), and eliminating CsrA regulation of these genes results in broad changes to the expression of downstream networks.

To evaluate the role of CsrA as a regulator of intermediate regulatory hubs, we selected two CsrA functional targets representative of two different types of regulatory proteins: *evgA*, which encodes for the transcriptional activator EvgA, and *acnA*, which encodes for a post-transcriptional regulator, AcnA. We chose these targets as examples of how CsrA could expand its influence on gene expression by controlling regulators that act at two different layers of regulation (transcriptional and post-transcriptional). Furthermore, these represented two convenient case studies due to their known regulated processes and distinct phenotypes. Concretely, EvgA is implicated in resistance to mildly acidic pH [43] and osmotic stress [31], as well as in multidrug resistance [44], and AcnA is associated with adaptation to nutritional and oxidative stress [32,33].

**Figure 1:**
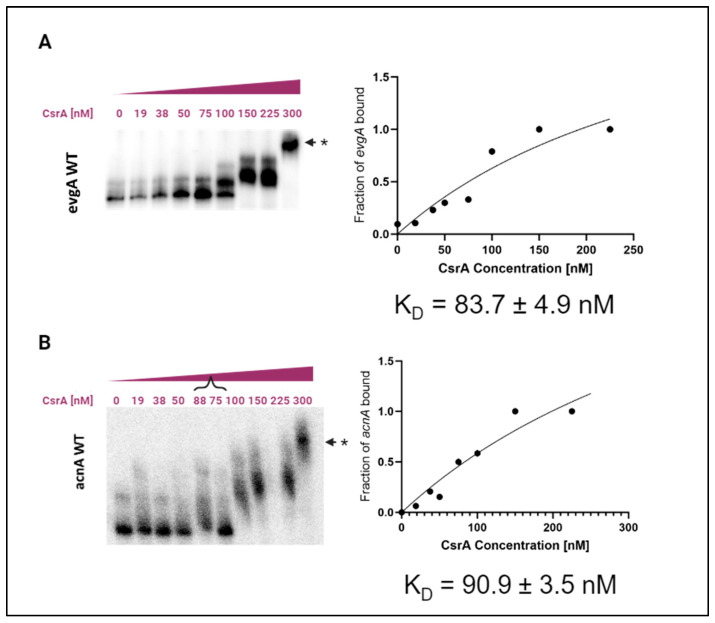
CsrA directly interacts with the *evgA* and *acnA* transcripts in vitro. 0.5 nM of P^32^-radiolabeled *evgA* and *acnA* were individually incubated with increasing concentrations of purified CsrA. All binding assays are performed in excess of yeast total RNA to inhibit the non-specific association of CsrA with labeled mRNA. (**A**) Electrophoretic mobility shift analysis (EMSA) of CsrA-binding to the *evgA* leader sequence. K_D_ was estimated from the fitted binding curve shown right. The standard deviation and 95% confidence intervals were determined from the non-linear fit of the individual gel measurements for each CsrA concentration. (**B**) EMSA of CsrA-binding to *acnA* and its respective fitted binding curve. Lanes 5 and 6, corresponding to 88 and 75 nM CsrA, respectively, were loaded in reverse order of the concentration gradient. These lanes are denoted with a bracket “{” for clarity. Supershiftted complexes that form at higher CsrA concentrations are indicated with an (*).

We first evaluated direct CsrA-binding to both the *evgA* and *acnA* mRNA transcripts using in vitro electrophoretic mobility shift assays (EMSAs). Specifically, 0.5 nM of P^32^- radiolabeled RNA was incubated with increasing concentrations of CsrA. Binding reactions were conducted as described by Yakhnin et al. (2012) [36] and others, and the formation of CsrA-RNA bound complexes was assessed using native polyacrylamide gel electrophoresis (PAGE). Similar to previous studies, the leader sequences (consisting of the annotated 5′ UTR and the first 100 nt of coding sequence) of *acnA* and *evgA* were used to evaluate binding, since CsrA is known to predominantly bind to the 5′ UTR and first nucleotides of coding sequence of its target RNAs; the exact sequences used for these experiments are listed in Supplementary Table S5 under “T7-acnA-WT” and “T7-evgA-WT”. From these assays, we determined CsrA to bind *evgA* with an apparent K_D_ ~ 83.7 ± 4.9 nM (Figure 1A) and to *acnA* with an apparent K_D_ ~90.9 ± 3.5 nM (Figure 1B). These values are on par with previously reported K_D_ values of CsrA-binding in *E. coli*, which range from 4 to 95 nM depending on the target RNA [12,21]. Higher order species (marked with an *) were observed at 300 nM CsrA, suggesting that CsrA potentially can simultaneously bind to more than one mRNA copy (Figure 1). Importantly, CsrA-*evgA* and CsrA-*acnA* interactions were successfully recapitulated in vivo using a plasmid-based fluorescence complementation assay previously developed in our lab [45,46] and shown to capture true CsrA interactions [4,22]. For *evgA*, we detected a 60% increase in YFP fluorescence signal relative to the *phoB* negative control, indicating successful YFP complementation that generated a signal above the background. Similarly, a 30% increase in fluorescence signal was observed relative to the *phoB* negative control when evaluating the interaction between CsrA and *acnA* (Supplementary Figure S1).

**Figure 2:**
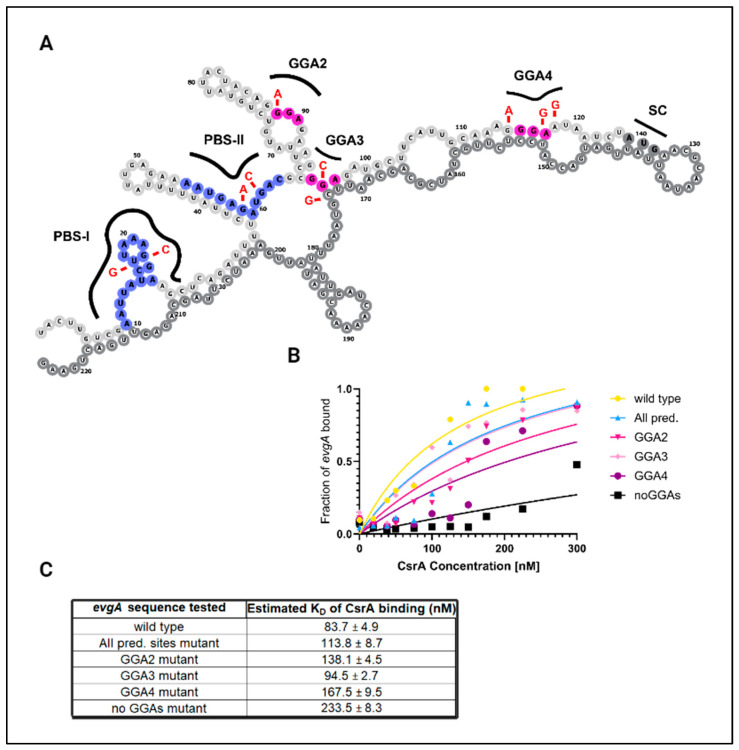
High-affinity GGA motifs mediate the CsrA-*evgA* interaction. (**A**) Secondary structure of the *evgA* leader sequence (5′ UTR + first 100 nt of coding sequence). The predicted binding sites from Leistra et al. (2018) [23] are shown in blue (PBS-I & PBS-II). PBS-I contains a high-affinity GGA motif. Additional GGA motifs that were considered for analysis are colored pink (GGA2, GGA3, and GGA4). The start codon (labeled “SC” in gray) and the coding sequence nucleotides are outlined in dark gray. Mutations introduced to test each individual binding site are shown in red. (**B**) Binding curves were generated via EMSAs for mutant versions of *evgA* to assess the contribution of each site to CsrA-binding, with (**C**) estimated K_D_ values determined from the binding curves. In this figure, “All pred. sites mutant” refers to a mutant of both PBS-I and PBS-II) and “no GGAs mutant” refers to a mutant of all GGA motifs.

**Figure 3:**
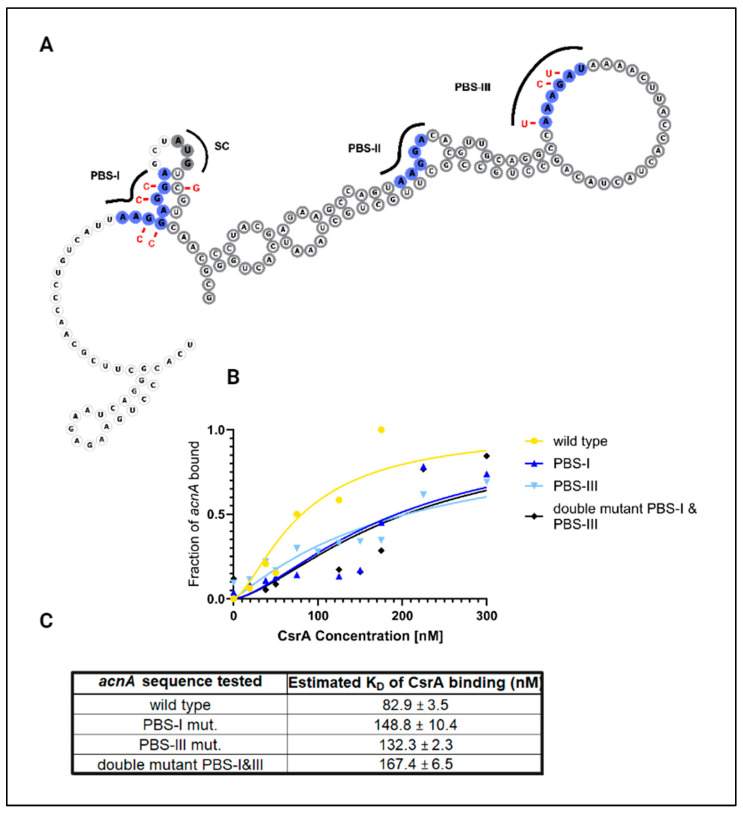
One high-affinity GGA motif and a degenerate ANGGN site mediate the CsrA-*acnA* interaction. (**A**) Secondary structure of the *acnA* leader sequence (5′ UTR + first 100 nt of coding sequence). The predicted binding sites from Leistra et al. (2018) [23] are shown in blue (PBS-I, PBS-II, and PBS-III). PBS-I and PBS-II contain high-affinity GGA motifs. The start codon (labeled “SC” in gray) and the coding sequence nucleotides are outlined in dark gray. (**B**) Binding curves were generated via EMSAs for mutant versions of *acnA* to assess their contribution of CsrA-binding, with (**C**) estimated K_D_ values determined from the binding curves.

To identify CsrA binding sites within *evgA* and *acnA*, we applied a mutational analysis approach combined with in vitro EMSAs (Supplementary Methods, Supplementary Table S5). Within the *evgA* sequence, thermodynamic modeling identified two binding pockets (defined as long stretches of overlapping ANGGN-like sites) within the 5′UTR of *evgA* as potential CsrA binding sites [23]. There are three additional GGA motifs in the 5′ UTR that were not predicted by the model but were selected for our mutational analysis since these are high-affinity motifs for CsrA-binding (Figure 2A). In the case of *acnA*, three GGA motifs (two located consecutively upstream of the start codon and one within the first 40 nucleotides of the coding sequence) were identified as potential CsrA binding sites from thermodynamic modeling by Leistra et al. (2018) [23]. An additional ANGGN-like sequence contained in a large, open loop in the coding sequence of *acnA* was identified as a potential CsrA binding site by the same model. In total, three potential binding sites in the *acnA* transcript were considered for mutational analysis: two corresponding to high-affinity GGA motifs and one corresponding to a non-consensus ANGGN-like sequence (Figure 3A). 

In our mutational analysis, potential CsrA binding sites were mutated individually and collectively, and changes in the equilibrium binding constant (K_D_) were determined via EMSAs for the CsrA-*evgA* and CsrA-*acnA* interactions. When possible, GGA→GCA substitutions were made to each mRNA transcript since this mutation has been reported to be sufficient to eliminate CsrA-binding [5]. Complementary mutations were made to preserve the predicted secondary structure and base-pairing probability of the mRNA sequences. If a potential CsrA binding site was located at the coding region of the mRNA, synonymous mutations were made to preserve codon identity. Supplementary Figure S2 (for *evgA*) and Supplementary Figure S3 (for *acnA*) show detailed schematics of the mutations introduced in each tested mRNA variant and their resulting binding curves. 

We first mutated the potential CsrA binding sites in the *evgA* leader predicted by Leistra et al. (2018) [23], which included one GGA motif in the first predicted binding site (PBS-I) and a non-consensus ANGGA-like site (PBS-II). The resulting mutant (labeled as “All pred. sites mutant”) had a modest ~1.2-fold increase in the apparent dissociation constant (K_D_) for its interaction with CsrA, suggesting that there are other sites within the *evgA* leader that CsrA can interact with. We next mutated the other three GGA motifs in *evgA* which represent potential sites for CsrA-binding (labeled as GGA2, GGA3, and GGA4). Using this approach, we identified “GGA2”, corresponding to the second GGA motif within the 5′ UTR of *evgA*, and “GGA4”, which overlaps the Shine–Dalgarno sequence, as the most likely CsrA binding sites (Figure 2C). Mutating “GGA2” (GGA→AGA) leads to a ~1.6-fold increase in the K_D_ for the CsrA- *evgA* interaction, while mutating “GGA4” (AGGGAA→AAGGGG) increases the K_D_ by ~2-fold. Importantly, while this work was in revision, Gorelik et al. (2024) also identified GGA4 as a CsrA-binding site within *evgA* [47]. Like other previously characterized direct CsrA targets, eliminating all the GGA motifs in the *evgA* sequence (no GGAs mutant) drastically reduces binding, as observed by the ~2.8-fold increase in K_D_ and the reduced amplitude of the binding curve (Figure 2B,C).

For *acnA*, mutating the GGA motifs of a potential binding site (GGAGGA→CCACCA) located upstream of the start codon (herein referred to as PBS-I) results in a ~1.7-fold increase in K_D_. Similarly, mutating the non-consensus ANGGN-like sequence (AAAGAU→UAACUU) in the coding sequence (herein referred to as PBS-III) increases the K_D_ by ~1.6-fold (Figure 3B,C). When both the upstream GGA motifs and the non-consensus ANGGN-like site (binding sites PBS-I and PBS-III) were mutated simultaneously (GGAGGA/AAAGAU→CCACCA/UAACUU), we observed a cumulative increase of ~2-fold in K_D_. From these results, we concluded that CsrA interacts predominantly with binding sites PBS-I and PBS-III of *acnA*. The contribution of both sites to CsrA-*acnA* binding was further supported by in vivo binding assays. In these assays, mutations at either PBS-I or PBS-III significantly impaired YFP complementation and led to a fluorescence signal that was indistinguishable from that of the *phoB* negative control (Supplementary Figure S4).

### 3.2. CsrA Represses Both evgA and acnA In Vivo and In Vitro

**Figure 4:**
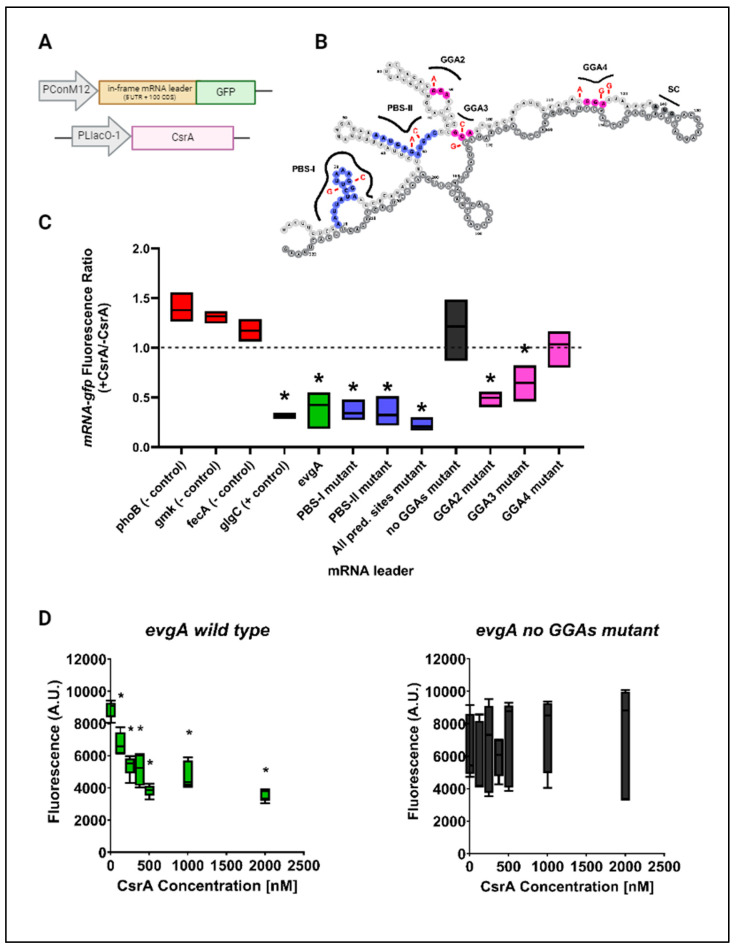
CsrA mediates *evgA* repression. (**A**) Diagram of the *evgA-gfp* in vivo reporter system. The first plasmid has CsrA expressed under the control of an IPTG-inducible promoter, while the second plasmid contains a constitutively expressed *evgA* leader sequence fused in-frame to *gfp*. (**B**) The secondary structure of the *evgA* leader with the predicted binding sites (blue) and additional putative GGA motifs (pink) is shown for reference. (**C**) Fluorescence ratios were calculated by dividing *evgA-gfp* fluorescence in the presence of CsrA by the fluorescence in the absence of CsrA. The results are representative of three independent biological replicates. Statistically significant values (*p*-value ≤ 0.05) are indicated by an asterisk (*) and were determined by comparing the negative control values to those of each mRNA variant tested using an unpaired *t*-test. (**D**) In vitro transcription–translation reactions were performed using the PURExpress kit with *evgA* wild-type and *evgA* no GGAs mutant translational fusions expressed from a T7 promoter. Increasing concentrations of purified CsrA were added prior to the start of each reaction. Values are shown for at least five replicates collected across three independent experiments. Statistically significant values are denoted with an asterisk (*) and indicate differences in fluorescence upon the addition of the respective CsrA concentration relative to the fluorescence when no CsrA was added to the reaction (0 nM). Figure created with BioRender.com.

From our mutational analysis and binding assays, we identified regions of *evgA* and *acnA* that contribute to CsrA-binding. In both mRNA targets, one of the likely CsrA binding sites is located near the start codon (i.e., “GGA4” in *evgA* and “PBS-I” in *acnA*). Traditionally, CsrA-binding at the Shine–Dalgarno sequence, or near the start codon, impedes ribosome binding and results in translational repression [48]. To investigate how CsrA impacts the expression of *evgA* and *acnA*, we applied an in vivo translational expression assay, previously developed by Adamson et al. (2013) [37] and adapted by Sowa et al. (2017) [22], to characterize CsrA-target interactions in vivo. In our experiments, the leader sequences of *evgA* and *acnA*, plus respective binding site mutants, were fused in-frame to a GFP reporter (Supplementary Methods, Supplementary Table S4). The plasmid containing our translational fusions was transformed into *E. coli* MG1655 K-12 Δ*csrABCD* Δ*pgaABCD* Δ*glgCAP* (herein referred to as Csr system deletion strain), together with a second plasmid containing CsrA under an IPTG-inducible promoter (Figure 4A), to enable controlling CsrA expressions. In the Csr system deletion strain, the *glgCAP* and *pgaABCD* operons are deleted to ensure cellular fitness upon the deletion of *csrA* [37]. Using this system, we compared the expression of our translational fusions in the presence and absence of CsrA. A previously developed *glgC-gfp* fusion reporter was used as a positive control [22]. Like previous observations using this reporter, the presence of CsrA (e.g., IPTG-induction of its expression) reduced the expression of the *glgC-gfp* fusion by ~2.5 fold, confirming its role as a repressor of *glgC*. Three negative control fusion reporters were also included in these experiments (*phoB-gfp, gmk-gfp*, and *fecA-gfp*). Minor changes in fluorescence upon CsrA induction were observed for all three reporters, confirming the lack of effect of CsrA presence in their expression. These changes were used to establish a baseline level and allowed us to differentiate between true activation and indirect effects upon induction with IPTG (Figure 4C).

In the case of *evgA*, CsrA induction reduced the expression of our translational reporter, indicating that CsrA-binding also represses *evgA* translation. This effect was alleviated only in the “GGA4” mutant and in the *evgA* mutant lacking GGA motifs, further indicating that “GGA4” is a preferred CsrA binding site and that CsrA binds *evgA* at high-affinity GGA motifs (likely “GGA2” and “GGA4”) (Figure 4C). To rule out indirect effects upon CsrA induction, we also assessed the regulatory effects of the CsrA-*evgA* interaction using an in vitro coupled transcription–translation PURExpress system (herein referred to as IVTT). In this system, the expression of an in-frame *evgA-gfp* fusion is driven by a T7 RNA polymerase promoter, and increasing concentrations of purified CsrA are added to the IVTT reactions. Consistent with our in vivo translational assays (Figure 4C), the expression of the *evgA-gfp* fusion was reduced upon the addition of CsrA, indicating that CsrA represses evgA translation (Figure 4D, left). Like the in vivo reporter, mutating all GGAs in the *evgA* leader sequence eliminated CsrA-dependent repression in vitro (Figure 4D, right). 

**Figure 5:**
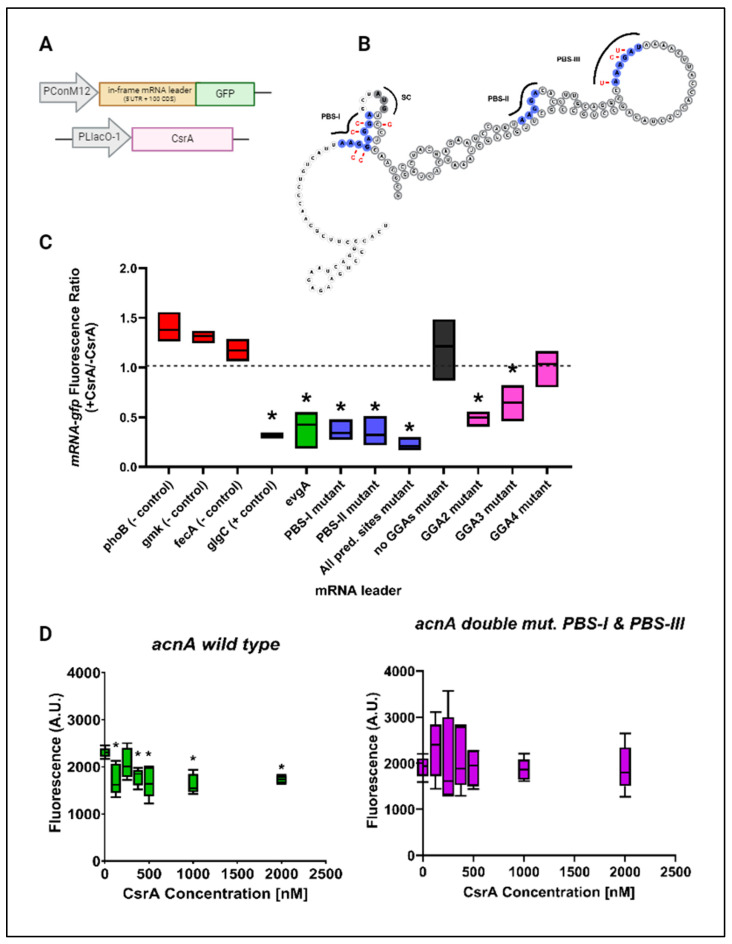
CsrA-mediated repression of *acnA*. (**A**) Diagram of the *acnA-gfp* in vivo reporter system. One plasmid contains CsrA expressed under the control of an IPTG-inducible promoter, while the second plasmid contains a constitutively expressed *acnA* leader sequence fused in-frame to *gfp*. (**B**) The secondary structure of the *acnA* leader with predicted binding sites (blue) considered for our mutational analysis is shown for reference. (**C**) Fluorescence ratios were calculated by dividing the *acnA-gfp* fluorescence in the presence of CsrA by the fluorescence in the absence of CsrA. The results are representative of three independent biological replicates. Statistically significant values (*p*-value ≤ 0.05) are indicated by an asterisk (*) and were determined by comparing the negative control values to those of each mRNA variant tested using an unpaired t-test. (**D**) In vitro transcription–translation reactions were performed using the PURExpress kit with *acnA* wild-type and *acnA* double mutant of PBS-I and PBS-III translational fusions expressed from a T7 promoter. Increasing concentrations of purified CsrA were added prior to the start of each reaction. Values are shown for at least five replicates collected across three independent experiments. Statistically significant values are denoted with an asterisk (*) and indicate differences in fluorescence upon the addition of the respective CsrA concentration relative to the fluorescence when no CsrA was added to the reaction (0 nM). Figure created with BioRender.com.

We observed repression of the *acnA-gfp* fusion upon CsrA induction in vivo (Figure 5C) using the same reporter system described above (Figure 5A). This effect was alleviated by mutating either binding site PBS-I or binding site PBS-III, suggesting that both sites are critical for CsrA-binding. The in vivo repression of *acnA* (Figure 5C) was recapitulated in vitro using the PURExpress IVTT system. In these in vitro reactions, the addition of CsrA resulted in a reduction in *acnA* translation (Figure 5D, left), but had no significant effect on the expression of the *acnA* double mutant-*gfp* fusion (binding sites PBS-I and PBS-III mutated simultaneously), further demonstrating that these two sites mediate the CsrA-*acnA* interaction (Figure 5D, right). It is worth mentioning that the repressive effects of CsrA in vitro are smaller than those observed in vivo. It is possible that the binding of CsrA in vivo induces structural changes that prevent additional enhancer factors, not present in the in vitro reactions, from binding the *acnA* leader, further repressing *acnA* translation in a cellular context. 

From these experiments, we concluded that CsrA binds *evgA* predominantly through the “GGA4” site and leads to translational repression, and that CsrA binds and represses *acnA* through the binding sites PBS-I and PBS-III. Notably, the binding site PBS-III of *acnA* is a non-consensus ANGGN-like site located at the coding sequence of the mRNA transcript. This finding expands the growing list of targets for which CsrA-binding occurs through a combination of a high-affinity GGA site and a degenerate, non-consensus ANGGN motif, as previously reported [4,9]. While the use of degenerate binding sites might be a more prevalent phenomenon among CsrA targets, only a few examples of these types of sites contributing to CsrA-binding have been characterized to date.

### 3.3. CsrA Influences Distinct Stress Phenotypic Clusters through Its Interactions with evgA and acnA

To investigate how CsrA-binding affects the respective gene target networks of EvgA and AcnA, we generated *evgA* and *acnA* genomic mutants in which CsrA binding sites were mutated to significantly reduce or eliminate the interactions between CsrA and these transcripts (Materials and Methods, Supplementary Table S2). We anticipated that this approach would allow us to capture the indirect targets of CsrA whose regulation is primarily mediated through *evgA* or *acnA*, since only the CsrA interaction with these central nodes would be impaired. To analyze transcriptional changes associated with strains with defective/abolished CsrA-*evgA* and CsrA-*acnA* interactions, we extracted RNA from genomic mutant strains and wild-type *E. coli* MG1655 K-12 at three growth phases (Early Exponential (OD_600_~0.8), Mid-Exponential (OD_600_~1.5), and Stationary phase (OD_600_~2.5)) (Supplementary Figure S5) to observe the effects of altering CsrA-binding over a broader window of time. 

**Figure 6:**
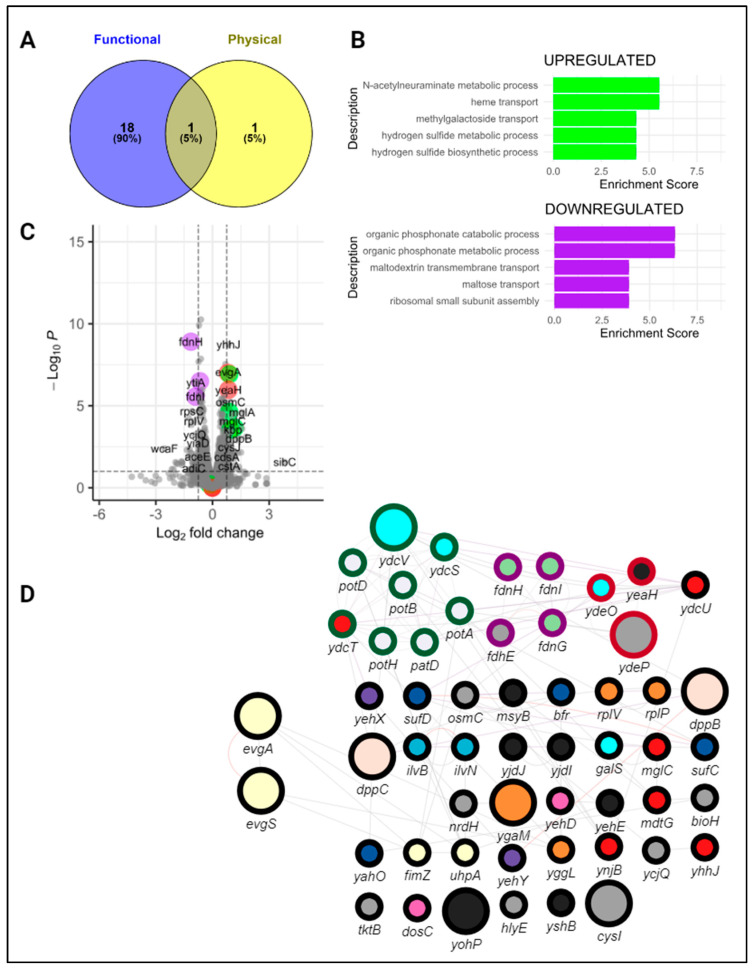
The expression of specialized gene clusters is influenced by the CsrA-*evgA* interaction. (**A**) Number of differentially expressed genes previously associated with CsrA. (**B**) Volcano plot of the differentially expressed genes in the *evgA* no GGAs genomic mutant relative to wild-type *E. coli.* Genes have colored outlines based on their cluster association in the gene network shown below (green: polyamine transport, purple: formate oxidation, and red: acid resistance). (**C**) EnrichGO analysis results showing the top 5 most upregulated and downregulated genes in the *evgA* genomic mutant at Early Exponential. (**D**) Network representation of the differentially expressed genes upon disruption of the CsrA-*evgA* interaction. Node size represents the number of growth conditions in which a gene was differentially expressed. Outlined colors denote genes clustered together in our network analysis. Genes with similar functions based on their GO annotations are filled with the same color.

After conducting RNA-seq analysis to compare gene expressions in the *evgA* genomic mutant relative to the wild-type *E. coli* MG1655 K-12 (parent strain), we identified a total of 36 genes as differentially expressed (at least 0.75 log2 fold-change; P_adj_ cutoff of <0.05) across the three growth conditions; 34 out of these 36 genes were differentially expressed during early exponential growth (Supplementary Table S6). Interestingly, while some of these genes (19/36) have been previously reported to be functionally altered by CsrA (i.e., CsrA affected their expression as determined via RNA-seq of a CsrA defective (*csrA::kan*) strain or using reporter assays), no evidence of direct binding has been reported in most cases (i.e., they have not been consistently detected in pull-down studies; only *ydcT* and *cysI*) (Figure 6A, Supplementary Tables S6 and S8). These observations indicated that CsrA regulation of these targets is primarily indirect and coordinated through the EvgA regulatory hub. 

**Figure 7:**
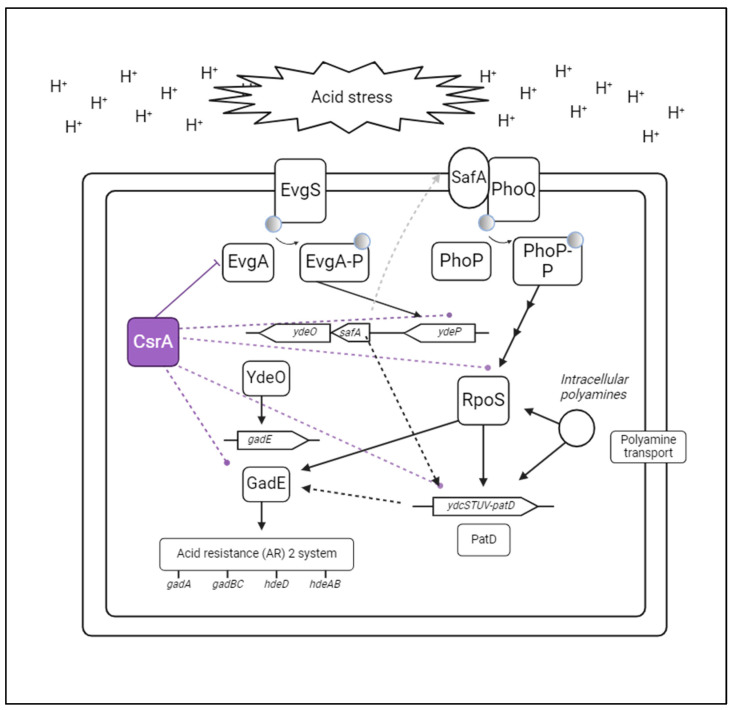
CsrA indirectly coordinates acid stress responses through its interaction with *evgA*. Schematic of known EvgA-regulated acidic stress-related genes. Solid lines denote known interactions. Light gray dotted lines indicate genes regulated by EvgA for which direct interactions are not known. Purple dotted lines indicate genes that are known to be functionally regulated by CsrA. Created with BioRender.com.

We next identified biologically relevant patterns and functional relationships between differentially expressed genes in the *evgA* genomic mutant strain via cluster analysis. Three clusters of functionally related genes were identified (Figure 6D), corresponding to acid resistance (outlined in red), formate oxidation (purple), and polyamine transport (green). EvgA-mediated acid resistance is relatively well understood. In response to changes in pH, EvgA transcriptionally activates the *ydeP-safA-ydeO* operon which activates the central regulator of the acid resistance 2 (AR2) system, GadE, through YdeO and a secondary activation route via the PhoP/Q two-component system (Figure 7) [43]. Our results suggest that *ydeP-safA-ydeO*, and *gadE* are functional targets of CsrA that are indirectly affected through its direct interaction with *evgA*. Formate oxidation is relevant to maintaining pH homeostasis and proton balance [49], and polyamines are stress molecules that promote the synthesis of proteins of the AR2 system [50,51], suggesting that the three clusters identified in this work together contribute to the cellular response to acid stress. While a direct link between EvgA and the genes in these last two clusters has not been established, they are likely part of the EvgA-coordinated response to acidic stress [52,53]. We further conducted enrichment analysis as a second approach to identify processes influenced by the CsrA-*evgA* interaction (Figure 6C). Using the enrichGO function of clusterProfiler [54,55] on our list of differentially expressed genes, we identified additional significantly enriched processes such as the metabolism of sialic acids (which is key for bacterial adaptation in the human gut, [56]), protein sulfydration (which plays an important role during cell signaling, [57]), organic phosphonate metabolism, and ribosomal assembly, which could all be involved in the mechanisms of EvgA-mediated multidrug resistance [58].

**Figure 8:**
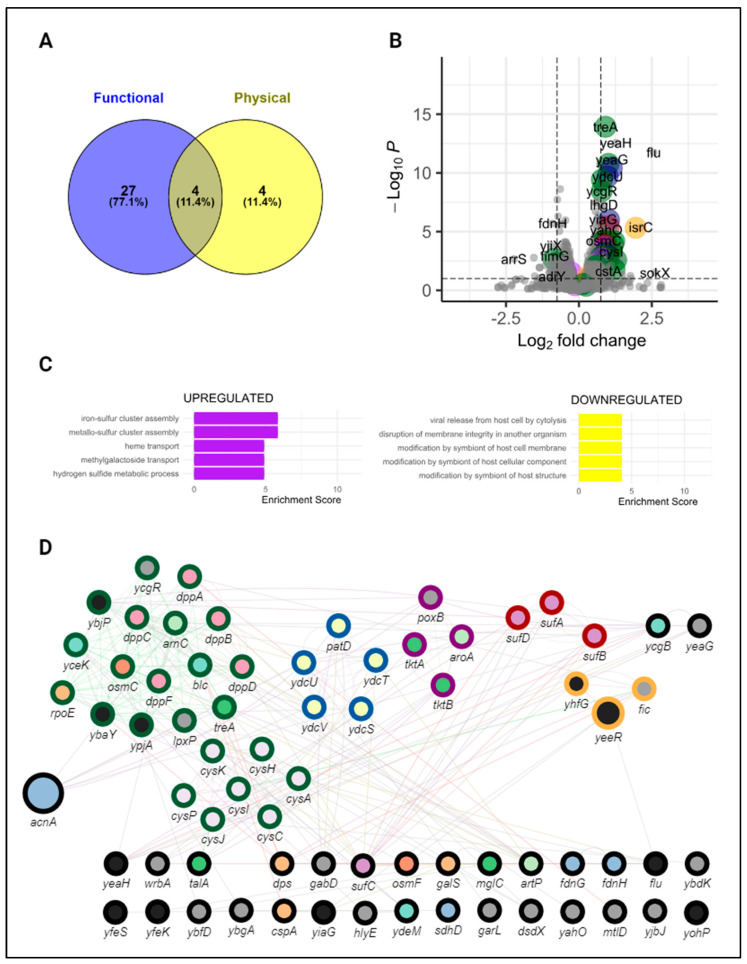
The CsrA-*acnA* interaction affects specialized gene clusters. (**A**) Number of differentially expressed genes previously associated with CsrA. (**B**) Volcano plot of the differentially expressed genes in the *acnA* genomic mutant (with mutated CsrA binding sites) relative to wild-type *E. coli*. Genes have colored outlines based on their cluster association in the gene network shown below (green: metal ion stress and DNA damage; blue: putrescine transport; purple: *poxB* and *tktB* cluster; red: iron–sulfur cluster; and yellow: toxin–antitoxin). (**C**) EnrichGO analysis results show the top 5 most upregulated and downregulated genes in the *acnA* genomic mutant at Early Exponential. (**D**) Network representation of the differentially expressed genes upon disruption of the CsrA-*acnA* interaction. Node size represents the number of growth conditions in which a gene was differentially expressed. Outlined colors denote genes clustered together in our network analysis. Genes with similar functions based on their GO annotations are filled with the same color.

**Figure 9:**
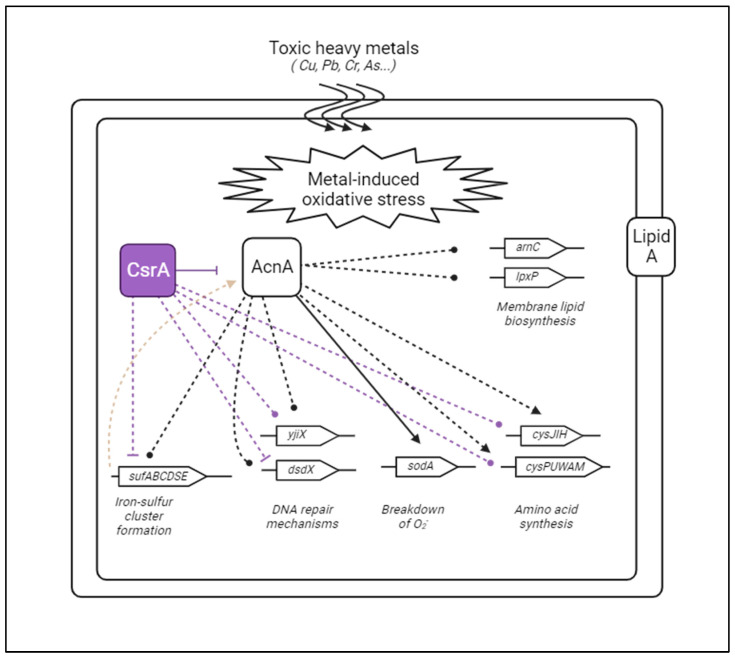
The CsrA-*acnA* interaction coordinates response systems for metal-induced oxidative stress. This simplified diagram illustrates the different pathways affected by CsrA through its interaction with *acnA* based on the observed differential expression of genes upon breaking the CsrA- *acnA* interaction. Solid lines denote known interactions. Light gray dotted lines indicate genes regulated by AcnA (inferred from RNA-seq) for which direct interactions are not known. Purple dotted lines indicate genes that are known to be functionally regulated by CsrA. Created with BioRender.com.

In parallel, upon disruption of the CsrA-*acnA* interaction, we observed that a total of 58 genes were differentially expressed by contrasting gene expression in the CsrA-*acnA* genomic mutant strain to that of wild-type *E. coli.* Similarly, to the *evgA* mutant, most genes (54/58) were previously observed to have a differential expression at early exponential growth (Supplementary Table S7). Over half of these genes have been functionally linked to CsrA (31/58) in the past, although evidence suggesting direct, physical CsrA-RNA interactions has only been reported for eight of them (*rpoE, osmF, IdtE, dps, sdhD, yjbJ, treA,* and *cysI*) (Figure 8A, Supplementary Tables S7 and S9). Our clustering analysis identified five unique clusters within differentially expressed genes (Figure 8D): one related to iron–sulfur cluster assembly during oxidative stress (outlined in red), one encompassing two homologs of a toxin–antitoxin system (*fic-yhfG*) (yellow), one including the *poxB* and *tktB* genes (which have been detected to be up-regulated at the protein level under aerobic, high-osmolarity conditions [59]) (purple), one corresponding to the *ydcSTUV-patD* operon (involved in putrescine transport, [60]) (blue), and a large cluster of genes involved in metal ion stress responses and DNA damage (green). Enrichment analysis by gene ontology further identified metal ion-related pathways as the most significantly enriched biological processes upon disruption of the CsrA-*acnA* interaction. (Figure 8C). This was particularly interesting, given that high concentrations of metal ions, and in particular heavy metals, are known to elicit both oxidative stress [61] and osmotic stress [62] responses in bacteria. The known role of AcnA as an RBP during oxidative stress [33], together with the differential expression of genes related to oxidative and osmotic stress adaptation, suggests a role for AcnA as a central regulator of metal ion responses and adaptation (Figure 9); in this proposed network, CsrA indirectly influences metal stress-related pathways through its regulation of *acnA*. Altogether, our findings illustrate a mechanism in which CsrA can achieve widespread control of stress responses through direct interactions with intermediate regulatory nodes (such as *evgA* and *acnA*).

### 3.4. The CsrA-evgA Interaction Is Important for Acidic Stress Tolerance in E. coli

**Figure 10:**
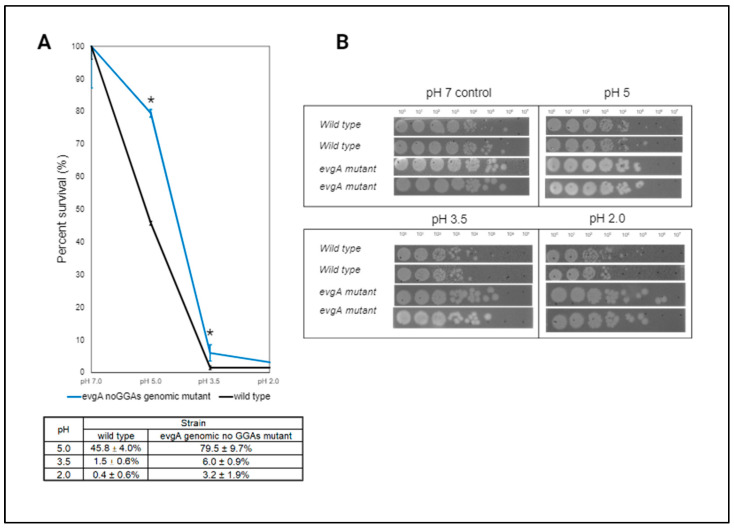
The interaction between CsrA and *evgA* impacts tolerance to acidic stress in *E. coli.* (**A**) Percentage of cell survival after a 2 h challenge at different pH values. The percentage was calculated by dividing the number of CFU/mL that grew post-stress treatment by the number of CFU/mL in an unstressed control. Error bars indicate percentage variations between independent biological triplicates. Asterisks denote statistically significant (*p*-value ≤ 0.05) differences between the percent survival of the *evgA* mutant (referred to as evgA genomic no GGAs mutant in the table) and that of wild-type *E. coli*. Significance was determined by contrasting the percent survival of these strains using an unpaired t-test. (**B**) Serial dilutions of the cultures post-stress were spot-plated to observe differences in acid stress tolerance. Images were collected for independent biological duplicates for each strain. Here, *evgA* mutant refers to a genomic mutant of all GGA motifs present in the *evgA* leader.

To investigate whether disrupting the CsrA-*evgA* interaction would generate biologically relevant phenotypes, we compared the survivability of the *evgA* genomic mutant of CsrA binding sites (labeled as *evgA* no GGAs genomic mutant in Figure 10) to that of wild-type *E. coli* K-12 MG1655 after being challenged with acidic conditions (pH = 2–5). The acid challenge was performed based on previous work by Nishino et al. (2003) [31] since acid stress responses are coordinated by the EvgA/EvgS two-component system. In these previous experiments, they observed that cells that lack the *acrAB* operon (*E. coli* KAM3) showed increased resistance to extremely acidic conditions when overexpressing *evgA* (7.9% survival compared to 0.1% survival for *E. coli* KAM3 at pH = 2.0). In our experiments, the cells were additionally challenged to mild (pH = 5.0) and extreme (pH = 3.5) acidic conditions since native induction of EvgA-regulated genes occurs at pH values of 5.5–4.5 [63], thereby suggesting that protective effects could potentially be observed at less extreme conditions (i.e., higher pH). As shown in Figure 10A, the CsrA-*evgA* genomic disruption mutant showed increased tolerance to acid stress, even under mildly acidic conditions. At pH 5, wild-*type E. coli* survivability was lower compared to that of the *evgA* genomic mutant (~40% reduction, *p*-value = 0.005). The enhanced survivability of the *evgA* genomic mutant strain was also observed at pH 3.5 (*p*-value = 0.002). Similar trends were observed when cells were spot-plated after the acidic challenge (Figure 10B), thereby demonstrating that the CsrA-*evgA* interaction impacts acid stress adaptation.

### 3.5. CsrA Influences Metal Ion Tolerance in E. coli through Its Interaction with acnA

**Figure 11:**
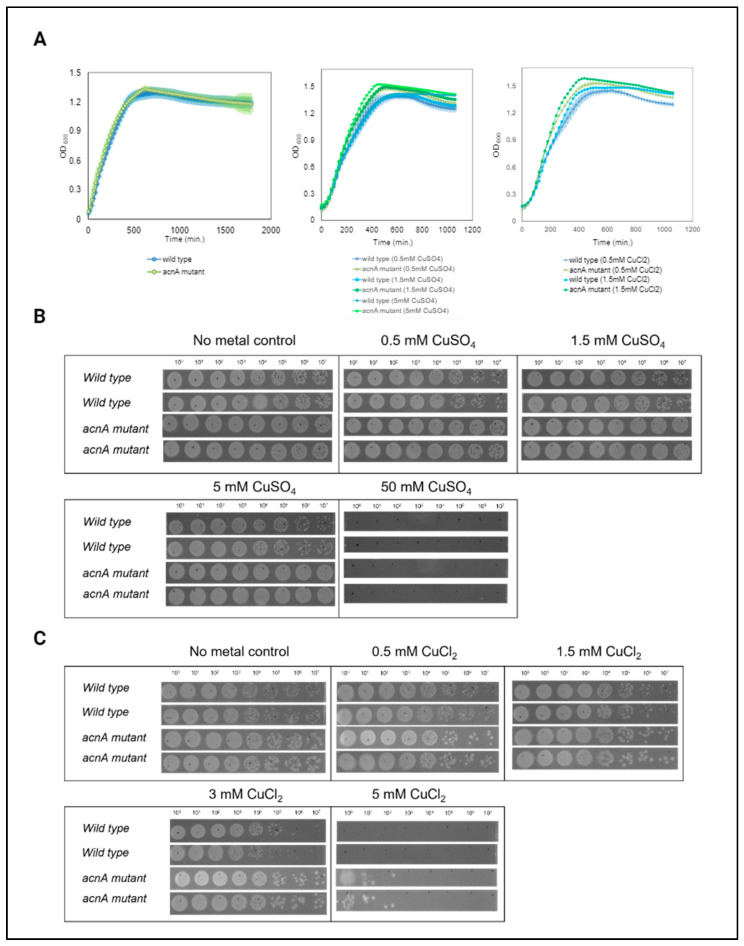
The CsrA-*acnA* interaction influences bacterial tolerance to heavy metal stress. (**A**) Growth curves of wild-type *E. coli* and *acnA* mutant (genomic mutant of CsrA binding sites in the acnA sequence) strains in LB media only (**left**) and LB media supplemented with increasing concentrations of CuSO_4_ (**middle**) and CuCl_2_ (**right**). Shading denotes the standard deviation between biological duplicates. Cells were challenged with increasing concentrations of (**B**) CuSO_4_ and (**C**) CuCl_2_ for 8 h. Serial dilutions of the cultures post-stress were spot-plated to observe differences in metal stress tolerance. Images represent the observed cell growth for independent biological duplicates of each strain.

RNA-seq results contrasting the expression patterns of an *acnA* genomic mutant (for which CsrA binding sites were mutated to disrupt the CsrA-*acnA* interaction) to those of wild-type *E. coli* suggest a central role for AcnA in mediating tolerance to heavy metals by regulating metal-induced oxidative stress and osmotic stress response processes (Figure 9). Previous work has shown that AcnA, in its inactive form, is an RNA-binding protein that contributes to adaptation during stationary growth and oxidative stress [34]. To evaluate if disrupting the CsrA-*acnA* interaction would result in a change in bacterial tolerance to metal stress, we examined the growth of the *acnA* genomic mutant strain in the presence of increasing concentrations of CuSO_4_ or CuCl_2_ and compared it to the growth of wild-type *E. coli* when exposed to the same concentrations of these metals. Both mutant and wild-type *E. coli* showed normal, exponential growth under ≤5 mM CuSO_4_ and ≤1.5 mM CuCl_2_. The *acnA* genomic mutant, however, had slightly improved tolerance to both metals (~10% increase in growth, *p*-value < 0.05 for CuSO_4_ after challenged for 8 h; ~7% increase in growth, *p*-value < 0.05 for CuCl_2_ after challenged for 8 h). These differences are apparent after eight hours of metal stress challenge (Figure 11A, middle and right panels). To better capture these differences, the cells were spot-plated in serial dilutions eight hours post-stress. The *acnA* genomic mutant was unaffected by CuSO_4_, as indicated by the equal cell density in unstressed and stressed cells. Wild-type *E. coli* was slightly more affected than the mutant, particularly when challenged with 5 mM CuSO_4_ (Figure 11B). Differences in growth were more apparent when cells were challenged with CuCl_2_. Increased tolerance by the *acnA* genomic mutant was evident at both 3 mM and 5 mM CuCl_2_ (Figure 11C), indicating that the CsrA-*acnA* interaction negatively controls adaptation to heavy metal-induced stress and validating the role of AcnA as an intermediate regulator of metal ion response systems. 

Cells were additionally challenged with increasing concentrations of Mg^2+^ since elevated concentrations of this ion can also be toxic to the cell and create ionic and osmotic stress [64,65]. While both strains grew similarly when supplemented with MgCl_2_ (Supplementary Figure S6A, middle), significant differences were observed with the addition of increasing concentrations of MgSO_4_. Concretely, at 0.5 and 1 M MgSO_4_, the *acnA* genomic mutant had a significantly increased tolerance to stress during exponential growth (Supplementary Figure S6A, right). Similar trends were observed when spot-platting the cells after being challenged with MgSO_4_ for 8 h (which is immediately before the timepoint at which we observed cells plateau and start declining in the 30 h growth curves) (Supplementary Figure S6B). However, at 0.5 M MgCl_2_, the wild-type strain showed increased tolerance compared to the *acnA* genomic mutant in these experiments. We attributed the enhanced tolerance to high MgSO_4_ concentrations to AcnA-mediated regulation of osmotic response systems. Likewise, the difference in tolerance when using the metal chloride or metal sulfate could be explained by differences in the intracellular uptake of these salts [66].

## 4. Discussion

CsrA is a global regulatory protein that influences numerous biological processes in *E. coli*. It has been proposed that CsrA may expand its regulatory influence over gene expression by directly binding and controlling other regulatory genes (such as those encoding for transcription factors or other regulatory proteins) which in turn control additional sub-specialized gene networks. However, confirmation of direct interactions between CsrA and these regulatory genes in vivo, as well as a mechanistic understanding of the regulatory outcome upon CsrA-binding, is yet to be elucidated in most cases. 

In this work, we have validated direct interactions between CsrA and two mRNA targets, *evgA* and *acnA* (Figure 1), which are repressed upon CsrA-binding (Figure 4 and Figure 5). Importantly, these two genes encode for regulatory proteins (a transcriptional regulator and an RNA-binding protein, respectively), which influence the expression of additional gene networks. As such, we hypothesized that *evgA* and *acnA* represent intermediate regulatory hubs that allow CsrA to extend its control over global gene expression. Notably, after identifying the regions of *evgA* and *acnA* that mediate CsrA-binding (Figure 2 and Figure 3) and generating genomic mutants with disrupted CsrA-*evgA* and CsrA-*acnA* interactions, we observed differential expression of many genes that have been previously proposed to be functionally affected by CsrA (Figure 6 and Figure 8). These results support the role of *evgA* and *acnA* as intermediate regulatory hubs for CsrA regulation. 

In the case of EvgA, we found that it allows CsrA to indirectly coordinate genes involved in acidic stress responses. This was further demonstrated by the enhanced tolerance of the *evgA* genomic mutant strain to this stress (Figure 10). Within the EvgA-mediated response to acid stress, RNA-seq results suggest that *ydeP-safA-ydeO* and *gadE* (which are functionally affected by CsrA) are indirect CsrA targets coordinated through the direct binding and regulation of the *evgA* transcript. The response to acidic stress is further facilitated via EvgA-mediated activation of the PhoP/Q two-component system which activates GadE through RpoS, another previously identified functional target of CsrA [67,68]. A schematic summarizing this regulatory circuitry is shown in Figure 7. Genes of the polyamine transport (*ydcSTUV-patD*) and formate oxidation (*fdnGHI*) clusters represent interesting candidates to further characterize the CsrA-*evgA* regulatory network and better understand the systems level response to acid stress in *E. coli*. While we did not investigate the impact of the CsrA-*evgA* interaction in multidrug tolerance, the identification of several enriched pathways related to antibiotic resistance suggests a potential regulatory role for CsrA indirectly affecting these processes through *evgA*. 

For AcnA, disrupting the CsrA-*acnA* interaction resulted in differential expression of genes related to metal-induced oxidative and osmotic stress. This suggested a central role of AcnA in mediating the adaptation to high concentrations of metal ions, and of particularly heavy metals that create oxidative stress. This role and the influence of CsrA over these processes through its interaction with *acnA* was further validated by phenotypic assays showing increased survivability of the *acnA* genomic mutant when challenged with increasing concentrations of metal ions (Figure 9 and Figure 11). Importantly, genes participating in these processes that were found to be differentially expressed upon disruption of the CsrA-*acnA* interaction are potential direct targets of AcnA, and further evaluation of their interactions would significantly expand our understanding of AcnA-mediated regulation. A simplified depiction of this network based on our findings is shown in Figure 9.

While the use of *acnA* and *evgA* genomic mutants depleted of CsrA binding sites allowed us to capture genes that are predominantly indirect CsrA targets, it is plausible that some of these genes could also be direct CsrA targets (which would suggest regulation redundancy). For instance, *rpoE* is a characterized direct target of CsrA which was found to be differentially expressed upon disruption of the CsrA-*acnA* interaction. Future work evaluating the target overlaps of the CsrA, AcnA, and EvgA regulatory proteins and the hierarchy of interactions will bring novel insights into the regulatory circuitry between their regulatory networks. It is interesting to think about how the dynamic regulation of mRNA transcripts might be achieved by simultaneously exerting direct and indirect regulation (i.e., via intermediate regulatory hubs for efficient genome-wide phenotypic shifts under stress).

Overall, we identified the network of genes affected by CsrA through their interactions with *acnA* and *evgA* and characterized relevant phenotypes for each network. Importantly, these results exemplify how CsrA can globally control gene expression through a combination of direct and indirect effects; this can represent a mechanism by which global regulatory proteins can have a broader effect in efficient post-transcriptional cell rewiring under stress. Recently, the *sra, bdm*, and *raiA* were identified as indirect CsrA targets [21], suggesting that the regulation of intermediate regulators by CsrA plays a larger role in expanding the regulatory network of this protein and that additional cases exist beyond EvgA and AcnA. Potentially, CsrA regulation of these regulators can alter the active pool of regulatory proteins—like in the case of regulators with overlapping putative targets that can work in concert or antagonize each other—that interact with target genes under specific conditions. How global post-transcriptional regulatory proteins, like CsrA, orchestrate combinatorial effects of regulatory interactions is an outstanding question that could be addressed with the development of integrative approaches to simultaneously interrogate multiple regulatory networks.

## Data Availability

All RNA-seq data referenced in this text have been uploaded to the SRA database and can be publicly accessed via BioProject number PRJNA1069781.

## Supplementary Materials

The following supporting information can be downloaded at: https://www.mdpi.com/article/10.3390/microorganisms12040636/s1, Supplementary Table S1.List of functional CsrA targets. Supplementary Table S2. evgA and acnA genomic mutants generated to hinder CsrA-mediated regulation. Supplementary Table S3. List of strains used in this study. Supplementary Table S4. List of plasmids and cloning techniques used in this study. Supplementary Table S5. List of oligos used in this study. Supplementary Table S6. DESeq2 analysis of transcriptomics experiment constrasting the evgA genomic mutant strain to the wild type strain. Supplementary Table S7. DESeq2 analysis of transcriptomics experiment constrasting the acnA genomic mutant strain to the wild type strain. Supplementary Table S8. Summary of differentially expressed genes in evgA genomic mutant. Supplementary Table S9. Summary of differentially expressed genes in acnA genomic mutant. Supplementary Information. Figure S1. CsrA binds to *evgA* and *acnA* in vivo. Direct protein-RNA binding was evaluated using a three-component fluorescence complementation assay. The leader sequence of the mRNA of interest is fused to the MS2 binding domain, the *rrnB* terminator, an MS2-linker-CYFP protein fusion, and a CsrA-linker-NYFP fusion. Direct CsrA-mRNA binding results in complementation of the YFP protein generates a fluorescence output. Fluorescence values are presented as the median of five independent biological replicates. Significantly higher fluorescence relative to the *phoB*-negative control indicates positive direct binding. Figure created with BioRender.com. Figure S2. Mutational analysis for the *evgA* leader sequence. The secondary structure of *evgA* was predicted using the Vienna RNA webserver. Mutations were designed to preserve the secondary structure and base-pairing probability of the overall structure. Binding sites considered for analysis (yellow) and the introduced mutations (red) are shown next to the structure. Figure S3. Mutational analysis for the *acnA* leader sequence. The secondary structure of *acnA* was predicted using the Vienna RNA webserver. Mutations were designed to preserve the secondary structure and base-pairing probability of the overall structure. Binding sites considered for analysis (yellow) and the introduced mutations (red) are shown next to the structure. For *acnA* wild type, lanes 5 and 6 were flipped with an image editor for consistency. Figure S4. In vivo binding assays support CsrA binding at PBS-I and PBS-III of *acnA*. Direct protein-RNA binding was evaluated using a three-component fluorescence complementation assay. (A) The leader sequence of *acnA* and different mutant versions were cloned into the pTriFC plasmid to test for in vivo CsrA-binding. (B) Direct CsrA-RNA binding results in complementation of the YFP protein, generating a fluorescence output. Fluorescence values are presented as the median of five independent biological replicates. Significantly higher fluorescence relative to the *phoB-negative* control indicates positive direct binding. Mutations to either PBS-I or PBS-III deterred YFP complementation, as evidenced by the reduction in YFP signal to background levels. Figure S5. Growth curves of *E. coli* MG1655 wild type, *acnA* and *evgA* genomic mutants. (A) Growth curves of *E. coli* K-12 MG1655 (parent strain, left), *E. coli evgA* no GGAs genomic mutant (middle), and *E. coli acnA* dm13 genomic mutant (right). The gray and black dots represent OD values measured by spectrophotometry for biological duplicates. The resulting fitted growth curves were used as input in a Baranyi bacterial growth model to obtain the growth parameters shown in (B). From the model result, samples representative of different growth phases were chosen for analysis. Specifically, the sample is at OD600~0.8 (Early Exponential), OD600~1.5 (Late Exponential), OD600~2.5 (Stationary). Superimposed growth curves are shown for reference: wild type (black), *evgA* genomic mutant (blue), and *acnA* genomic mutant (green). Figure S6. CsrA influences bacterial tolerance to metal stress through its interaction with *acnA*. (A) Growth curves of *E. coli* wild-type and *acnA* genomic mutant strains in LB media only (left) and LB media supplemented with increasing concentrations of MgCl_2_ (middle) and MgSO_4_ (right). Shading denotes the standard deviation between biological duplicates. (B) Cells were challenged with increasing concentrations of MgCl_2_ (top, middle, & right) and MgSO_4_ (bottom) for 8 h. Serial dilutions of the cultures post-stress were spot plated to observe differences in metal stress tolerance. References in the Supplementary Information Document are included in the list of References [69,70,71,72].
