# Supplementary material for: The Post-Transcriptional Regulatory Protein CsrA Amplifies Its Targetome through Direct Interactions with Stress-Response Regulatory Hubs: The EvgA and AcnA Cases
Source: Microorganisms. 2024 Mar 22;12(4):636. doi: 10.3390/microorganisms12040636 (PMC11052181; doi:10.3390/microorganisms12040636)
Supplement: Supplementary file 1 [file microorganisms-12-00636-s001.zip › Supplementary-Information-01262024.pdf]

Supplementary Information for

The post-transcriptional regulatory protein CsrA amplifies its targetome through direct interactions with stress-response regulatory hubs: the EvgA and AcnA cases.

Alejandra Matsuri Rojano-Nisimura[1], Kobe Grismore[2], Josie S. Ruzek[2], Jacqueline L. Avila[2], and Lydia M. Contreras[1,2,*]

1 Department of Molecular Biosciences, The University of Texas at Austin, Austin, TX6 78712, USA
2 McKetta Department of Chemical Engineering, University of Texas at Austin, 200 E. Dean Keeton St. Stop C0400, Austin, TX 78712, USA9

*Address correspondence to Lydia M. Contreras, lcontrer@che.utexas.edu.

Table of Contents

Supplementary Methods

- Protein purification
- Three-fluorescence complementation assay
- Acidic Stress Experiments
- Metal Ion Stress Experiments

Supplementary Figures

Supplementary Data

Supplementary Methods

Protein purification

CsrA was purified as previously published in [4]. Briefly, His-tagged CsrA (CsrA-H6) was expressed from a pET-21a (+) vector (pCSB12) in *E. coli* BL21(DE3). Following protein expression and cell lysis, CsrA was purified from the soluble fraction (~10 mL) via nickel column purification using a Ni-NTA agarose column resin (Qiagen). Bound CsrA was recovered with 50% elution buffer (250 mM imidazole) and exchanged into CsrA storage buffer (10 mM Tris-HCl, 100 mM KCl, 10 mM MgCl2, 25% glycerol, pH 7.0). Protein concentration was determined by Bradford assay and stored at -20°C.

Three-fluorescence complementation assay

Fluorescence assays were performed as described in [46] with slight modifications. The two-plasmids system (pTriFC: RNA-MS2 binding domain-rrnB fusion + CsrA-NYFP & pMS2-CYFP: MS2-linker-CYFP) was transformed into *E. coli* MG1655 K-12 Δ*csrB*. Biological quintuplet colonies were picked and grown to saturation in 5 mL starter cultures at 37ºC overnight. Fresh 5 mL subcultures were seeded (1:100 dilution, in flasks) and grown at 37ºC until mid-exponential (OD_{600}~0.4-0.6). Expression of the fusion constructs from pLacO promoters was induced with 1 mM IPTG (final concentration). Cultures were then grown at 37ºC for a total of 22 hours. Yellow fluorescence was measured in a LSRFortessa Flow Cytometer (BD Biosciences) and median fluorescence values were computed. Fluorescence medians were compared to that of the *phoB-MS2-rrnB* fusion which was used as a negative control by two-tailed heteroscedastic t-tests.

Acidic Stress Experiments

To evaluate the tolerance to acidic stress of our genomic *evgA* mutant, we performed acid challenge experiments as described by [31] with a few modifications. Mutant and wild type *E. coli* strains were grown overnight (~18-20 hrs) in LB medium. Overnight cultures were diluted 1:1000 into LB at different pH values (7, 5, 3.5 and 2). These values were chosen to evaluate mild acidic stress based on previous reports that EvgA-regulated genes are induced at pH values of 5.5-4.5 [62]. Acid challenge was carried out by incubating the cells for 2 hours at 37°C and shaking at 200 rpm. Afterwards, cells were serially diluted and plated for evaluating survival. The percentage of survival was calculated by dividing the number of CFU/mL remaining after acid challenge by the number of CFU/mL in the unstressed control. Serial dilutions were also spot platted to better capture differences in survivability between strains.

Metal Ion Stress Experiments

Tolerance to metal ion stress was evaluated by adapting a previously published protocol by [68] used to profile the response of *E. coli* to heavy metal stress. Specifically, cells were grown in 5 mL of Luria-Bertani (LB) medium (Benton-Dickenson and Company) at 37°C overnight (~20 hrs.). After that, overnight cultures were used to seed new cultures of 5 mL LB broth supplemented with $MgCl_2$ or $MgSO_4$(Fisher Scientific) at final concentrations of 0.1, and 0.5 M for each metal. Survivability at 1 M $MgSO_4$ was evaluated ($MgCl_2$ was not tested at 1 M since that concentration is more than the reported MIC for that metal). Concentrations were chosen to mimic low, mild, and high toxicity conditions based on previous work assessing survivability of *E. coli* after challenging them to metal ion stress [63,69,70]. Additionally, cells were challenged with increasing concentrations of $CuSO_4$ to evaluate survivability to heavy metals. Cultures were supplemented with $CuSO_4$ at final concentrations of 0.5, 1.5, 3, 5 and 50 mM based on previous work determining the MIC of *E. coli* for this metal [71]. Cells were simultaneously seeded in LB only media as an unstressed growth control. Cells were

adjusted to equal starting OD_{600}= 0.1. Afterwards, 200 µL of each cultured condition were used to fill a 100-well honeycomb plate (Fisher Scientific). Growth curve analysis was performed by incubating the plate in a Bioscreen C Type FP-1100-C Analysis System (Thermo Labsystems). Temperature was set at 37°C, with medium and continuous shaking. Wavelength measurements were taken at 600 nm every 20 minutes for a total incubation time of 30 hrs. For spot platting experiments, the same protocol was followed with the difference that cells were challenged in metal-supplemented medium for 8 hours at 37°C incubation with 200 rpm shaking. Afterwards, serial dilutions of each culture were used for plating.

Supplementary Figures

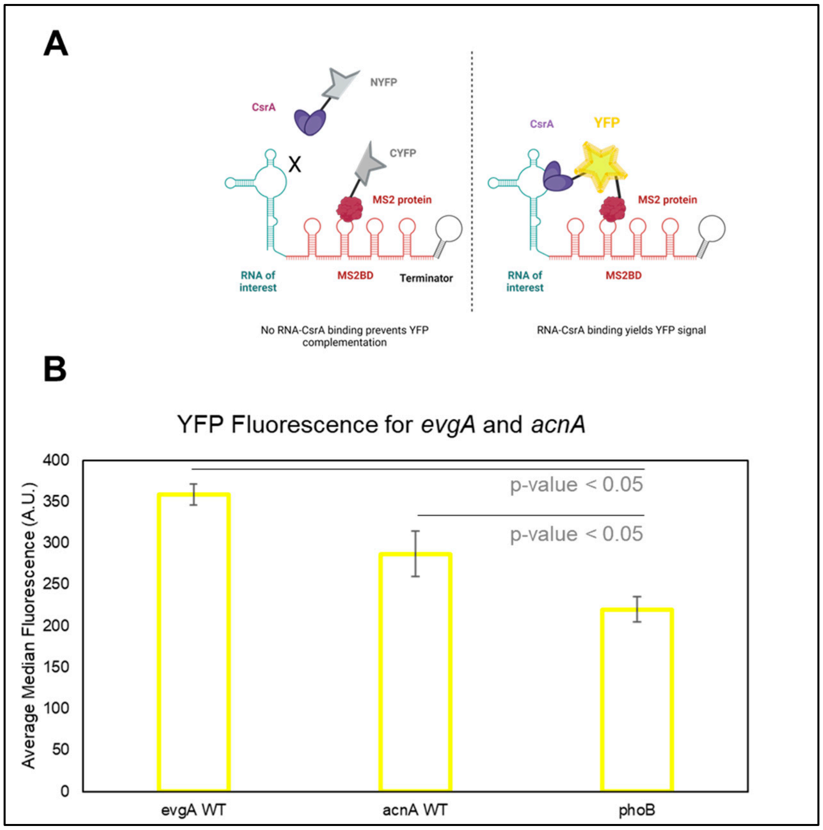

Supplementary Figure S1. CsrA binds to *evgA* and *acnA in vivo*. Direct protein-RNA binding was evaluated using a three-component fluorescence complementation assay. The leader sequence of the mRNA of interest is fused to the MS2 binding domain, the *rrnB* terminator, an MS2-linker-CYFP protein fusion, and a CsrA-linker-NYFP fusion. Direct CsrA-mRNA binding results in complementation of the YFP protein generates a fluorescence output. Fluorescence values are presented as the median of five independent biological replicates. Significantly higher fluorescence relative to the *phoB*-negative control indicates positive direct binding. Figure created with BioRender.com

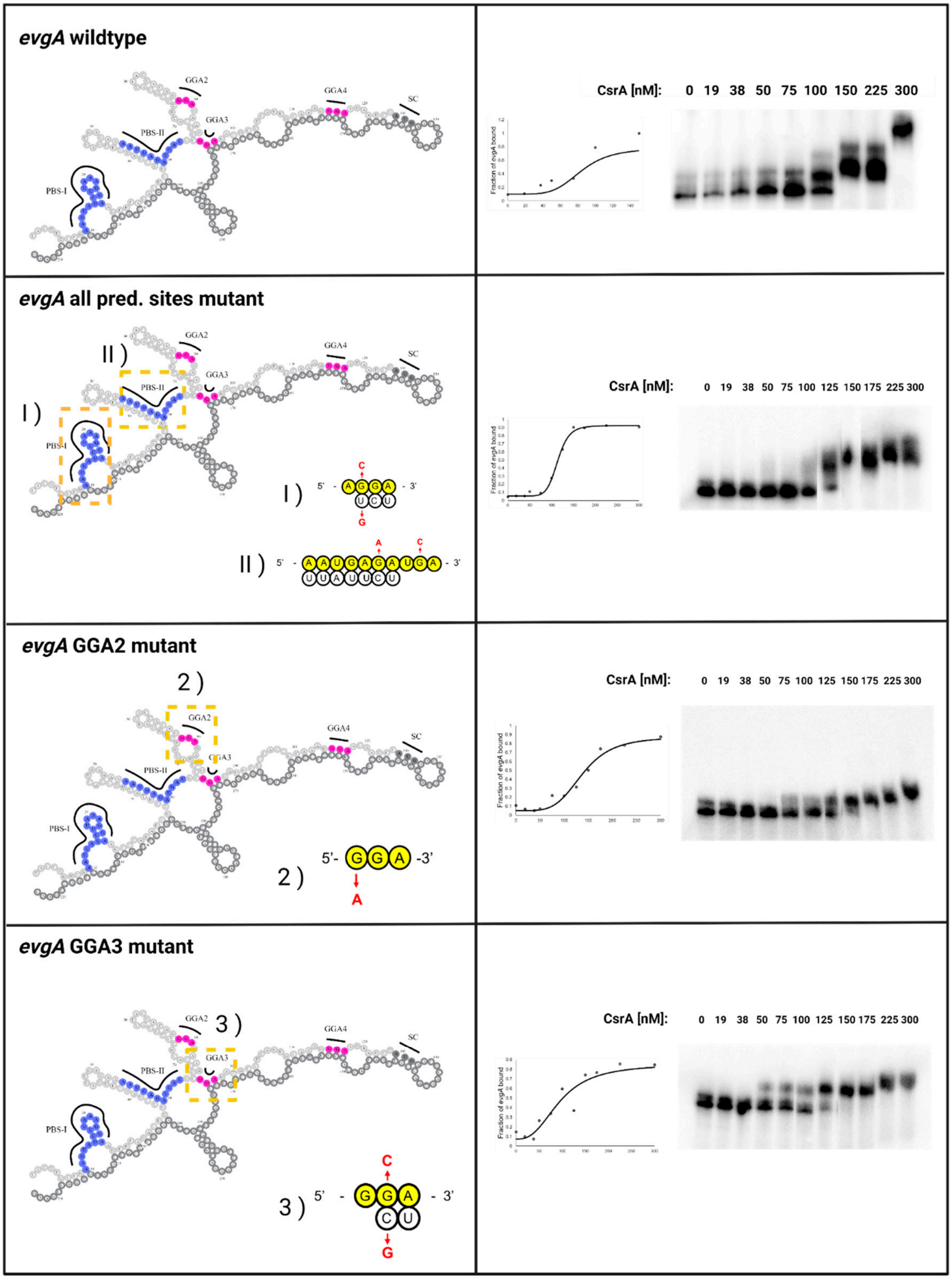

evgA wildtype
GGA2
GGA3
GGA4
SC
PBS-II
PBS-I
CsrA [nM]: 0 19 38 50 75 100 150 225 300
Fraction of evgA bound
evgA all pred. sites mutant
I)
II)
5' - AGGA - 3'
UCU
C
G
5' - AAUGAGAUGA - 3'
UUAUUCU
A
C
CsrA [nM]: 0 19 38 50 75 100 125 150 175 225 300
evgA GGA2 mutant
2)
5'- GGA -3'
A
CsrA [nM]: 0 19 38 50 75 100 125 150 175 225 300
evgA GGA3 mutant
3)
5' - GGA - 3'
CU
C
G
CsrA [nM]: 0 19 38 50 75 100 125 150 175 225 300

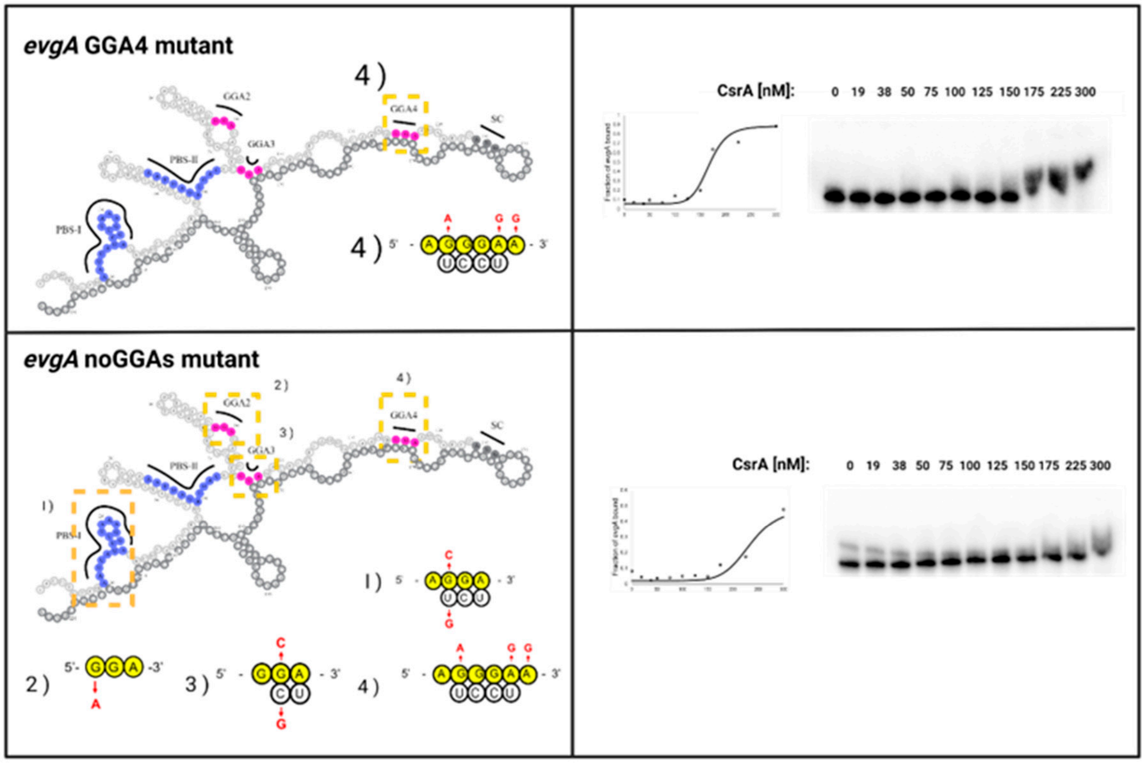

Supplementary Figure S2. Mutational analysis for the *evgA* leader sequence. The secondary structure of *evgA* was predicted using the Vienna RNA webserver. Mutations were designed to preserve the secondary structure and base-pairing probability of the overall structure. Binding sites considered for analysis (yellow) and the introduced mutations (red) are shown next to the structure.

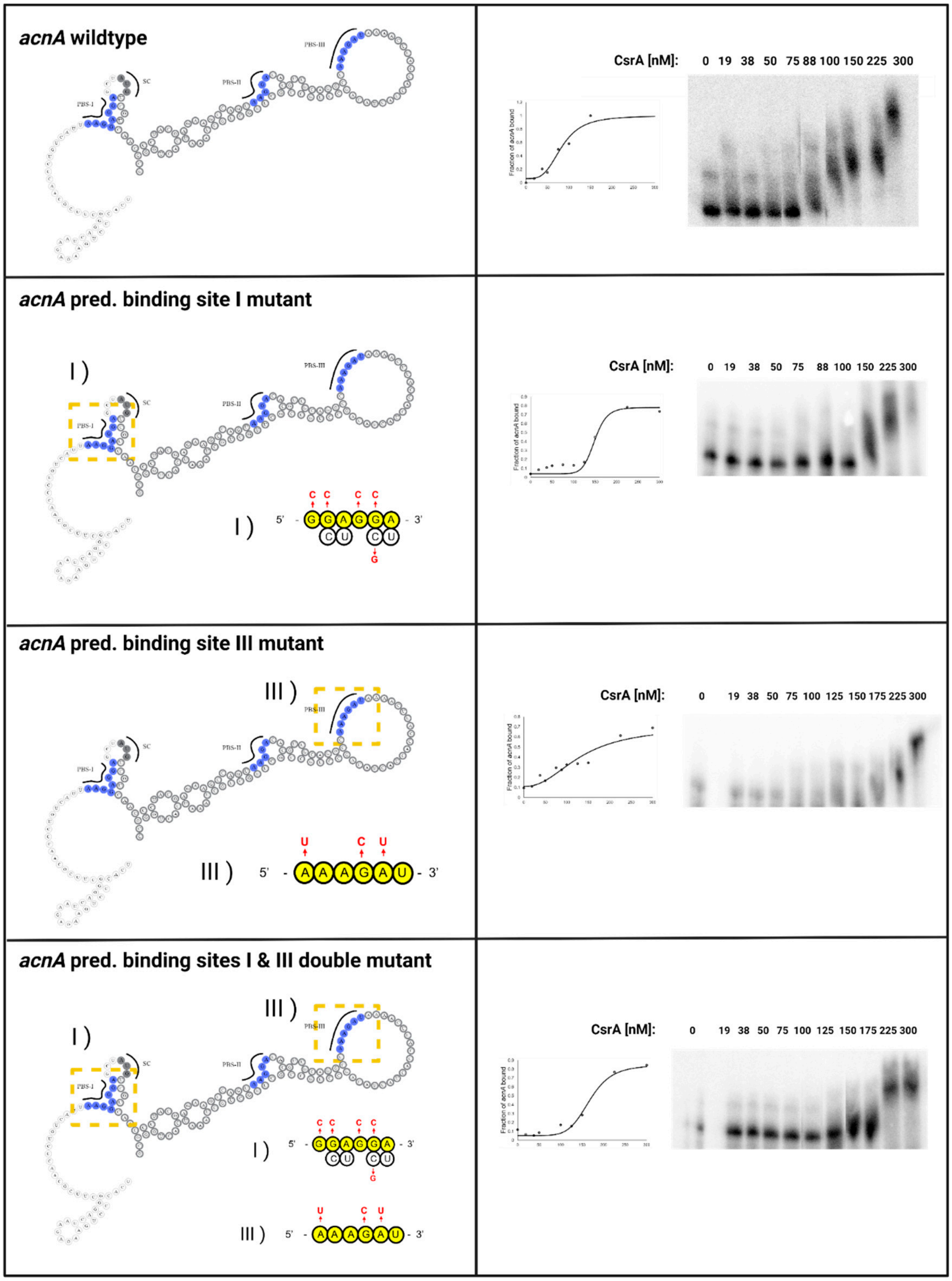

Supplementary Figure S3. Mutational analysis for the *acnA* leader sequence. The secondary structure of *acnA* was predicted using the Vienna RNA webserver. Mutations were designed to preserve the secondary

structure and base-pairing probability of the overall structure. Binding sites considered for analysis (yellow) and the introduced mutations (red) are shown next to the structure. For *acnA* wild type, lanes 5 and 6 were flipped with an image editor for consistency. The original image for this gel is included below as part of Supplementary Data.

A

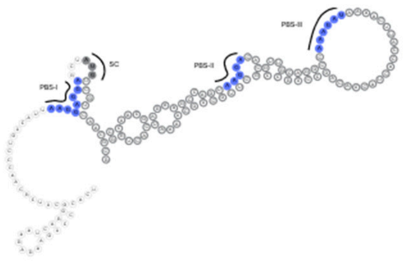

ID	Description
acnA WT	Wild type acnA sequence
acnA all pred. sites	acnA triple mutant w/ PBS-I, PBS-II & PBS-III mutated simultaneously
PBS- III	Predicted binding site 3 single mutant
PBS- II	Predicted binding site 2 single mutant
PBS- I	Predicted binding site 1 single mutant
PBS-II & PBS-III double mut.	Predicted binding sites 2 and 3 mutated simultaneously
PBS-I & PBS-II double mut.	Predicted binding sites 1 and 2 mutated simultaneously

B

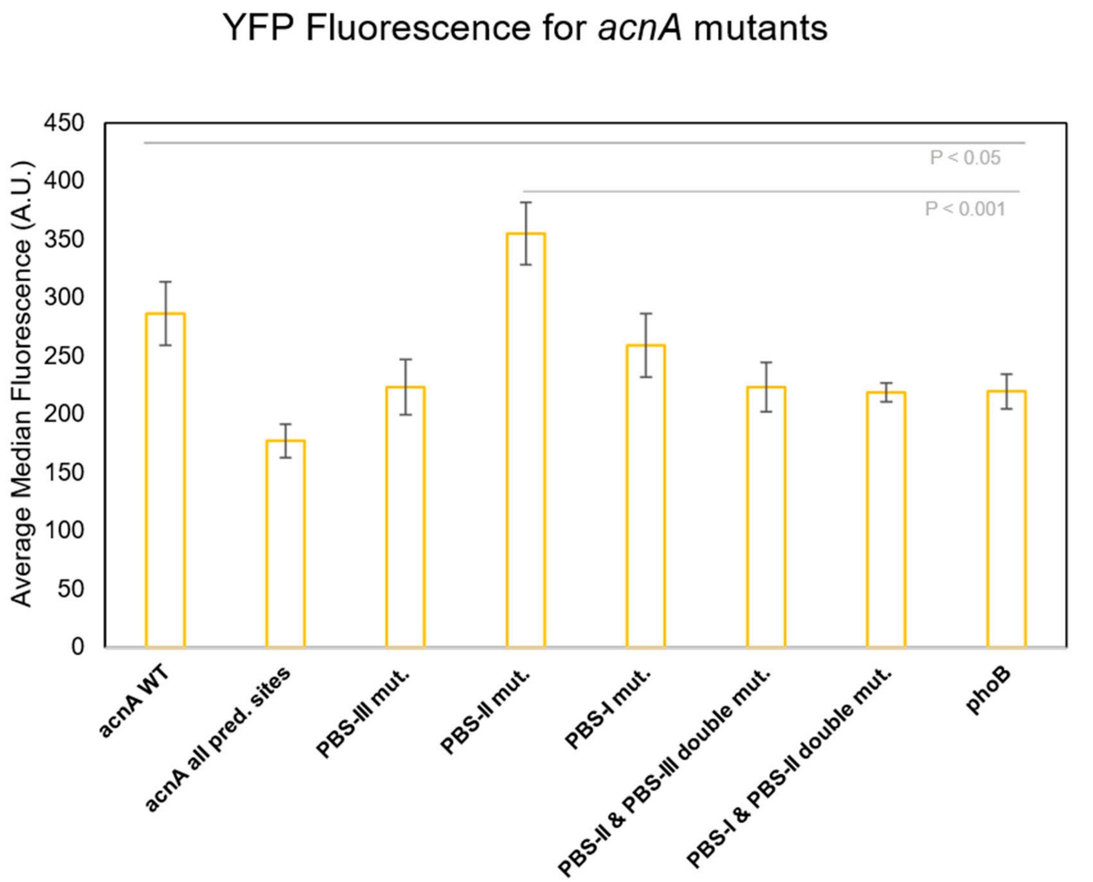

Supplementary Figure S4. *In vivo* binding assays support CsrA binding at PBS-I and PBS-III of *acnA*. Direct protein-RNA binding was evaluated using a three-component fluorescence complementation assay. (A) The leader sequence of *acnA* and different mutant versions were cloned into the pTriFC plasmid to test

for *in vivo* CsrA-binding. (B) Direct CsrA-RNA binding results in complementation of the YFP protein, generating a fluorescence output. Fluorescence values are presented as the median of five independent biological replicates. Significantly higher fluorescence relative to the *phoB-negative* control indicates positive direct binding. Mutations to either PBS-I or PBS-III deterred YFP complementation, as evidenced by the reduction in YFP signal to background levels.

A

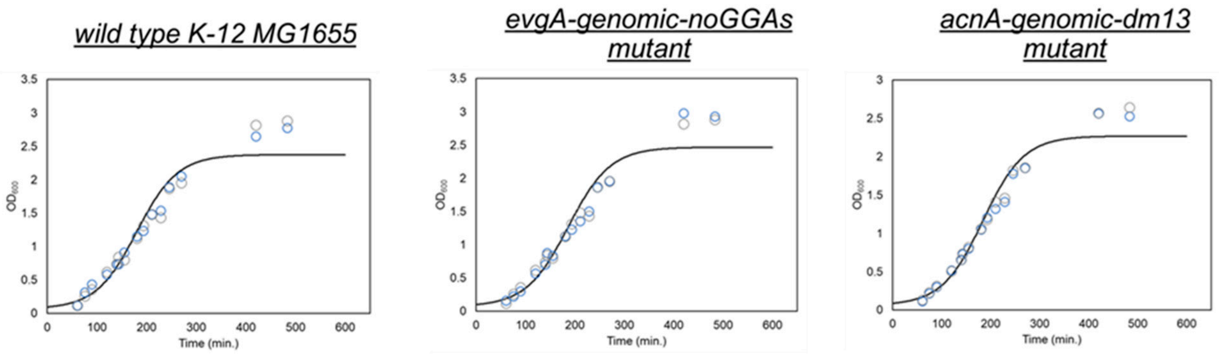

B

Strain	Lag phase	Max. exponential	Stationary
E. coli K-12 MG1655	OD_{600}<0.120	OD_{600}~0.8-1.5	OD_{600}>2.4
E. coli evgA genomic no GGAs mutant	OD_{600}<0.120	OD_{600}~0.8-1.5	OD_{600}>2.4
E. coli acnA genomic dm13 mutant	OD_{600}<0.120	OD_{600}~0.8-1.5	OD_{600}>2.3

C

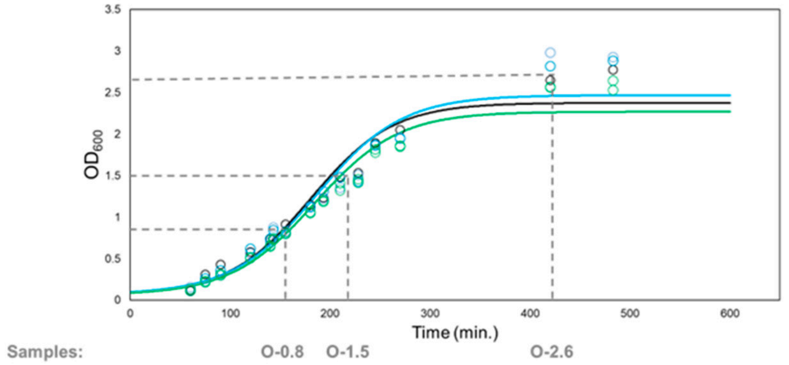

Supplementary Figure S5. Growth curves of *E. coli* MG1655 wild type, *acnA* and *evgA* genomic mutants. (A) Growth curves of *E. coli* K-12 MG1655 (parent strain, left), *E. coli evgA* no GGAs genomic mutant (middle), and *E. coli acnA* dm13 genomic mutant (right). The gray and black dots represent OD values

measured by spectrophotometry for biological duplicates. The resulting fitted growth curves were used as input in a Baranyi bacterial growth model to obtain the growth parameters shown in (B). From the model result, samples representative of different growth phases were chosen for analysis. Specifically, the sample is at OD600~0.8 (Early Exponential), OD600~1.5 (Late Exponential), OD600~2.5 (Stationary). Superimposed growth curves are shown for reference: wild type (black), *evgA* genomic mutant (blue), and *acnA* genomic mutant (green).

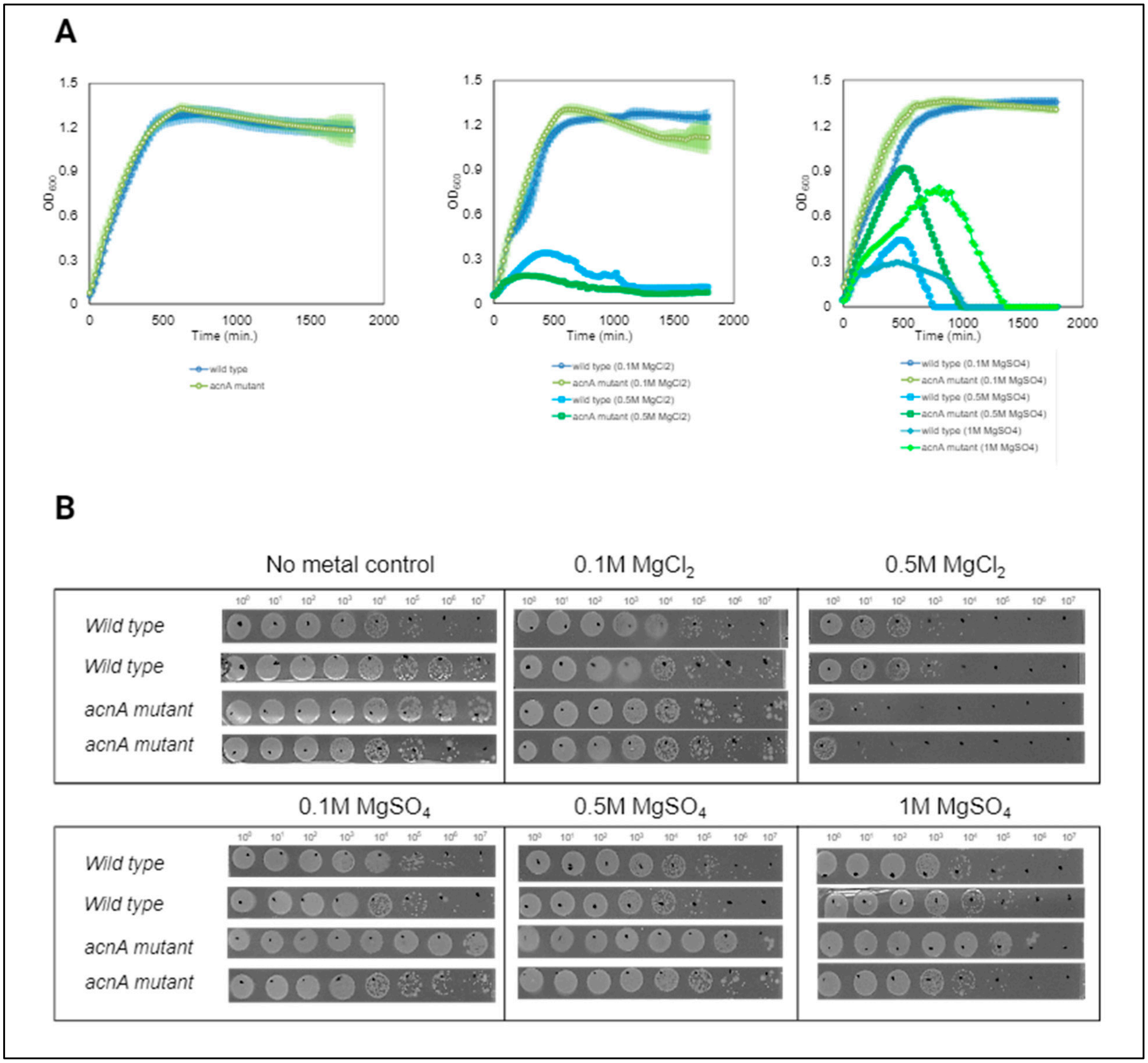

Supplementary Figure S6. CsrA influences bacterial tolerance to metal stress through its interaction with *acnA*. (A) Growth curves of *E. coli* wild-type and *acnA* genomic mutant strains in LB media only (left) and LB media supplemented with increasing concentrations of MgCl2 (middle) and MgSO4 (right). Shading denotes the standard deviation between biological duplicates. (B) Cells were challenged with increasing

concentrations of $MgCl_2$ (top, middle, & right) and $MgSO_4$ (bottom) for 8 hours. Serial dilutions of the cultures post-stress were spot plated to observe differences in metal stress tolerance.

<u>Supplementary Data</u>

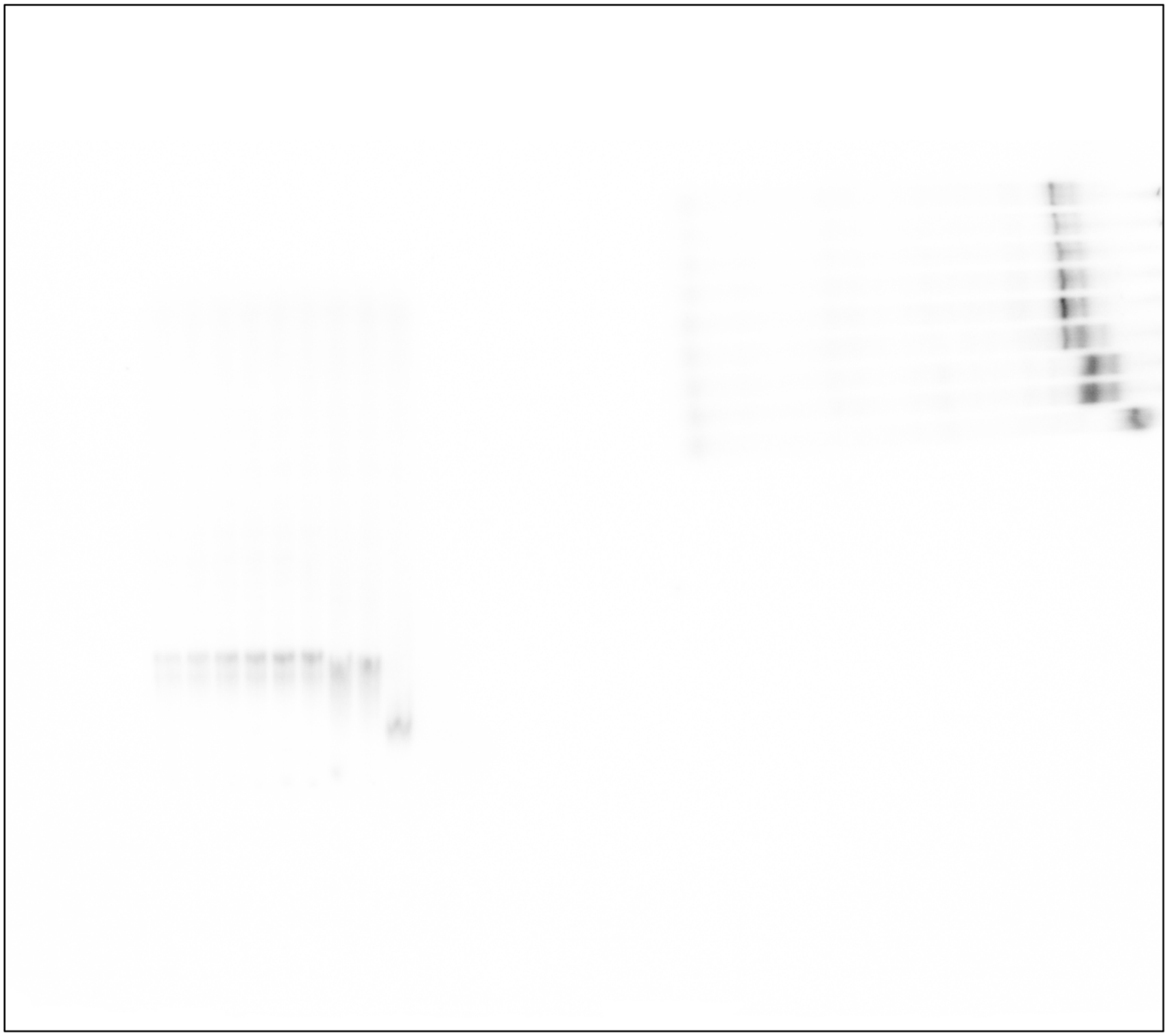

Unprocessed membrane image of CsrA-*evgA* EMSA gel. The corresponding processed image of this file is presented in the main text as part of Figure 1A. Top gel image was used to analyze the interaction between purified CsrA and T7 *in vitro* transcribed *evgA*. Bottom gel image was part of a different analysis not presented in this manuscript.

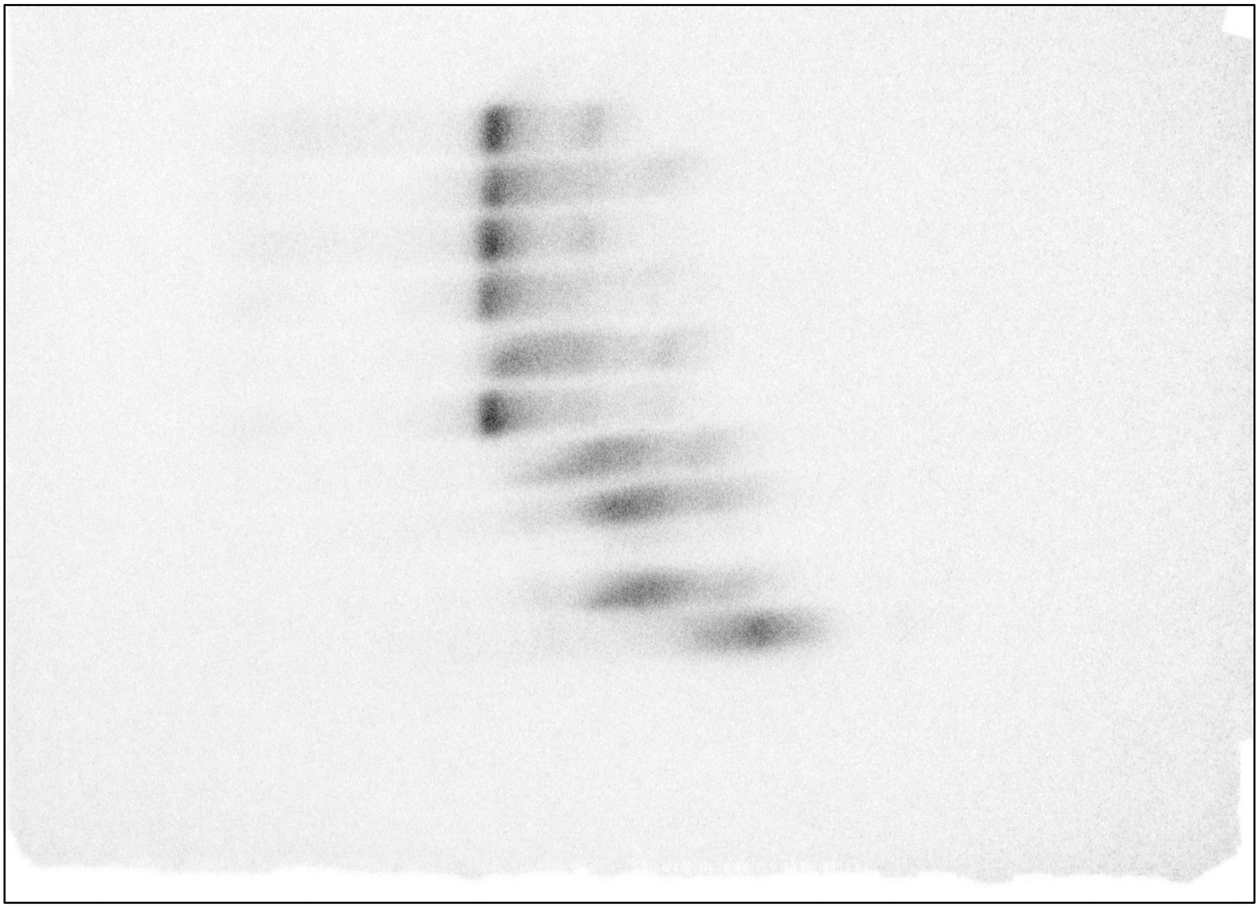

Unprocessed membrane image of CsrA-*acnA* EMSA gel. The corresponding processed image of this file is presented in the main text as part of Figure 1B.
